# Isolation and characterisation of a novel *Silviavirus* bacteriophage promising antimicrobial agent against methicillin-resistant *Staphylococcus aureus* infections

**DOI:** 10.1038/s41598-024-59903-w

**Published:** 2024-04-22

**Authors:** Varintip Lerdsittikul, Sukanya Apiratwarrasakul, Thassanant Atithep, Patoo Withatanung, Nitaya Indrawattana, Pornpan Pumirat, Somjit Chaiwattanarungruengpaisan, Metawee Thongdee

**Affiliations:** 1 Veterinary Diagnostic Center, Faculty of Veterinary Science, Mahidol University, Nakhon Pathom, Thailand; 2 Frontier Research Center, Vidyasirimedhi Institute of Science and Technology, Rayong, Thailand; 3 Department of Immunology, Faculty of Medicine Siriraj Hospital, Mahidol University, Bangkok, Thailand; 4 Department of Microbiology and Immunology, Faculty of Tropical Medicine, Mahidol University, Bangkok, Thailand; 5 The Monitoring Surveillance Center for Zoonotic Diseases in Wildlife and Exotic Animals, Faculty of Veterinary Science, Mahidol University, Nakhon Pathom, Thailand; 6 Siriraj Center of Research and Excellence in Allergy and Immunology (SiALL), Faculty of Medicine Siriraj Hospital, Mahidol University, Bangkok, Thailand

**Keywords:** Microbiology, Molecular biology

## Abstract

The increasing prevalence of methicillin-resistant *Staphylococcus aureus* (MRSA) emphasises the urgent need for novel antimicrobial agents as alternatives to antibiotics. Bacteriophage therapy is one of the most promising antimicrobial strategies. Here, we isolated and comprehensively characterized a novel *Staphylococcus* phage, vB_SauM_VL10 (VL10), from urban sewage. The VL10 genome displays 141,746 bp of linear double-stranded DNA, containing 193 open reading frames and lacking tRNA, virulence, or antibiotic resistance genes. Phylogenetic analysis categorizes VL10 as a novel species within the *Silviavirus* genus, *Twortvirinae* subfamily. VL10 exhibits lytic behaviour characterized by efficient adsorption, a short latent period, and substantial burst size, with environmental stability. It demonstrates lytic activity against 79.06% of tested *S. aureus* strains, highlighting its species specificity. Additionally, VL10 effectively targets MRSA biofilms, reducing biomass and viable cells. In MRSA-infected *G. mellonella* larvae, VL10 enhances survival rates, supporting its potential for phage therapy applications. Moreover, the emergence of VL10-resistant *S. aureus* strains associated with fitness trade-offs, including reduced growth, biofilm formation, and virulence. Altogether, these findings emphasize VL10 as a promising candidate for developing therapeutic agents against MRSA infections, providing insights into phage biology and resistance dynamics.

## Introduction

*Staphylococcus aureus*, an important Gram-positive bacterium, is recognized as a major cause of nosocomial infections worldwide^1,2^. This pathogen causes a variety of human diseases ranging, from mild infections including skin or soft tissue infections and abscesses to life-threatening conditions such as endocarditis, sepsis, purulent inflammation, and toxic shock syndrome^2,3^. Antibiotics are the treatment of choice for *S. aureus* infections, however, they can be challenging to administer due to the rising frequency of antibiotic-resistant strains, particularly methicillin-resistant *Staphylococcus aureus* (MRSA) in various countries, including Thailand^4^. MRSA exhibits resistance not only to beta-lactam antibiotics but also to other drug classes, including aminoglycosides, macrolides, fluoroquinolones, chloramphenicol, and tetracycline, leading to the emergence of multidrug-resistant *S. aureus* (MDRSA)^5^. Recognizing the urgency of the situation, the World Health Organization (WHO) has designated MRSA as a high-priority pathogen, necessitating the search for novel antibiotics and treatment strategies^6^. Furthermore, *S. aureus* can form biofilms that either adhere to native tissues or persist on the surface of medical devices, leading to antibiotic resistance and therapeutic failures, as biofilms protect the bacteria from antibiotics and the host immune system attacks^7,8^. Therefore, there is an urgent need to find antimicrobial alternatives to combat MRSA and its biofilm formation.

In response to these challenges, attention is turning toward alternative therapeutic options such as bacteriophage therapy. Bacteriophages (also known as phages) are a group of viruses that specifically infect and destroy bacterial cells and considered as a promising tool to eliminate multidrug-resistant bacteria and biofilm-associated infections^9,10^. Phages present several advantages over antibiotics: they are self-propagations, highly specific targeting infected bacterial cells without harming the normal microbiota, and relatively safe^11^. Phages ideal for therapeutic applications must be thoroughly evaluated in terms of their broad host range, life cycle, production capacity (large burst size), storage stability, the absence of undesirable genetic elements e.g., toxins, antibiotic-resistant or integrase genes and its anti-biofilm activity^12^. Numerous *Staphylococcus* phages have been discovered and characterised in both in vitro and in vivo studies, evaluating their potential as treatments for MRSA infections^13–16^. For instance, *Staphylococcus* phage ΦMR003 has demonstrated a broad host range and effective lytic activity against MRSA strains of human origin^17^. Additionally, *Staphylococcus* phage ΦSA012 has been found to enhance the survival rate of mice infected with MRSA, showing promise as a potential treatment option^16^. *Staphylococcus* phage SDQ has proven to be highly effective in disrupting MRSA biofilms and maintaining a long-lasting bacteriostatic effect, underscoring its potential for controlling MRSA biofilms in clinical and industrial applications^18^.

While *S. aureus*-specific bacteriophages have been discovered and isolated on a global scale, there has been a notable lack of emphasis on phages originating in Southeast Asia. It is critical to recognise that phages isolated from a single geographical region may have varying efficacy when applied to bacterial strains from different locations. As a result, research efforts have been focused primarily on the isolation and characterisation of phages native to specific regions, as well as their compatibility with autochthonous bacterial strains. Furthermore, the establishment of comprehensive national phage collections, similar to phage banks^19^, has become an urgent task. This pursuit is critical for the development of effective phage cocktail therapies as well as the advancement of our understanding of these invaluable biological resources.

In this study, we isolated and fully characterized a novel *Staphylococcus* phage named vB_SauM_VL10 (herein referred to as VL10) from sewage samples for its potential in MRSA control through phage therapy. The research included a genome sequence analysis, virion morphology, host range, phage adsorption, replication kinetics, and stability under varying temperature and pH conditions. Additionally, we examined the ability of VL10 to combat MRSA biofilms in vitro and its effectiveness in treating systemic MRSA infections in a *Galleria mellonella* model. Furthermore, the isolation and characterization of VL10-resistant *S. aureus* strains provided insights into the physiological trade-offs associated with phage resistance. These findings contribute to our understanding of phage biology and underscore VL10’s promise as a therapeutic agent against MRSA infections.

## Results

### Phage vB_SauM_VL10 isolation, morphology, and host range

**Figure 1:**
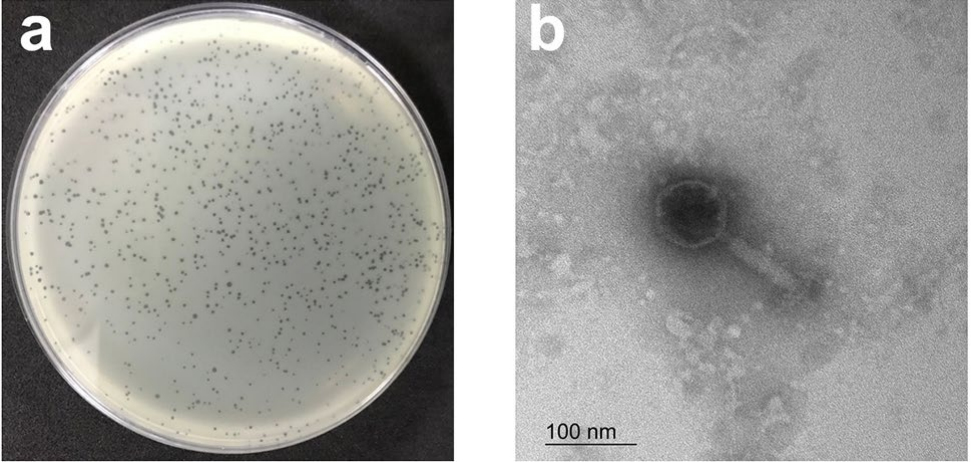
vB_SauM_VL10 morphology. (**a**) Plaque morphology of phage VL10 formed in a double-layer agar plate with MRSA ATCC 43300. (**b**) Transmission electron micrographs of phage VL10 negatively stained with 2% (w/v) uranyl acetate. The scale bar represents 100 nm.

*Staphylococcus* phage vB_SauM_VL10 (VL10) was isolated from samples of urban sewage collected in Bangkok, Thailand. The isolated phage formed round translucent plaques with a range of size 1–2 mm in diameter on medium containing lawns of MRSA ATCC 43300 (Fig. 1a). Transmission electron microscopy (TEM) examination indicated that phage VL10 had an icosahedral head with a diameter of 85.55 ± 6.10 nm and long contractile tails with a length of 136.70 ± 16.52 nm (*n* = 5 phages) (Fig. 1b).

The host spectrum of VL10 was determined on a panel of 43 *S. aureus* strains. This panel included standard laboratory strains and clinical isolates from either human or companion animal diseases (as listed in Table 1), with their respective antimicrobial susceptibility profiles presented in supplementary data, Table S1. Our investigation revealed that VL10 exhibited lytic activity against 79.06% (34 out of 43) of *S. aureus* strains tested. However, this phage did not exhibit lytic activity on other Gram-positive strains including *S. pseudintermedius*, Coagulase-negative *Staphylococci* used in this study (Table 1). These findings emphasize the highly specific nature of VL10 towards *S. aureus* and has a broad lytic spectrum within this species. Moreover, to semi-quantify the lytic activity of VL10, an efficiency of plating (EOP) experiment was performed on the 34 *S. aureus* strains susceptible to VL10. The results of EOP analysis revealed that 76.47% (26 out of 34) of the *S. aureus* isolates displayed high EOP values while 2.94% (1 out of 34) and 20.59% (7 out of 34) displayed medium and low values, respectively (Table 1).

**Table 1:** Host range and efficiency of plating (EOP) of phage vB_SauM_VL10 against standard laboratory strains and clinical isolates of *S. aureus* (MRSA or MSSA), *S. pseudintermedius* and Coagulase-negative *Staphylococci*.

Bacterial species	Strain	Source	Plaque formation	EOP
Standard laboratory strains of *S. aureus*	ATCC43300 (MRSA)	American type culture collection (ATCC)	+	High (host) (1 + 0.00)
	ATCC29213 (MSSA)	ATCC	+	High (2.95 ± 0.07)
	ATCC25923 (MSSA)	ATCC	+	High (1.2 ± 0.28)
	MRSA DMST20646	Department of Medical Sciences, Thailand (DMST)	−	ND
	MRSA DMST 20649	DMST	+	High (1.41 + 0.10)
	MRSA DMST 20651	DMST	+	High (1.22 + 0.11)
	MRSA DMST 20652	DMST	+	High (1.31 + 0.09)
Human-associated MRSA or MSSA Human-associated MRSA or MSSA	MSSA-TM1	Clinical isolation	+	High (1.25 ± 0.07)
	MRSA-TM2	Clinical isolation	+	High (1.1 ± 0.14)
	MSSA-TM3	Clinical isolation	−	ND
	MSSA-TM4	Clinical isolation	−	ND
	MSSA-TM5	Clinical isolation	−	ND
	MSSA-TM6	Clinical isolation	−	ND
	MSSA-TM7	Clinical isolation	−	ND
	MSSA-TM8	Clinical isolation	+	High (2.35 ± 0.49)
	MSSA-TM9	Clinical isolation	+	High (0.625 ± 0.03)
	MSSA-TM10	Clinical isolation	−	ND
	MSSA-TM11	Clinical isolation	+	Low (0.044 ± 0.04)
	MSSA-TM12	Clinical isolation	+	High (0.75 ± 0.07)
	MSSA-TM13	Clinical isolation	+	Low (0.0185 ± 0.02)
	MSSA-TM14	Clinical isolation	+	High (0.625 + 0.04)
	MSSA-TM15	Clinical isolation	+	Low (0.0045 + 0.001)
	MSSA-TM16	Clinical isolation	+	High (2.11 ± 0.15)
	MSSA-TM17	Clinical isolation	+	Moderate (0.34 ± 0.05)
	MSSA-TM18	Clinical isolation	+	Low (0.002 ± 0.001)
	MSSA-TM19	Clinical isolation	+	High (1.15 ± 0.21)
	MSSA-TM20	Clinical isolation	+	Low (0.08)
	MSSA-TM21	Clinical isolation	+	High (1.075 ± 0.106)
	MSSA-TM22	Clinical isolation	+	High (2.12 ± 0.169)
	MSSA-TM23	Clinical isolation	−	ND
	MSSA-TM24	Clinical isolation	+	High (1.95 ± 0.07)
	MSSA-TM25	Clinical isolation	+	High (0.745 ± 0.06)
	MSSA-TM26	Clinical isolation	+	Low (0.078 ± 0.01)
	MSSA-TM27	Clinical isolation	+	Low (0.084 ± 0.007)
	MSSA-TM28	Clinical isolation	+	High (3.1 ± 0.14)
	MSSA-TM29	Clinical isolation	+	High (2.2 ± 0.28)
	MSSA-TM30	Clinical isolation	−	ND
Animal-associated MRSA or MSSA	MSSA-279	Wound swab/cat	+	High (1.25 ± 0.35)
	MSSA-556	Wound swab/cat	+	High (0.95 ± 0.07)
	MRSA-461	Wound swab/cat	+	High (2.16 ± 0.23)
	MSSA-891	Urine/cat	+	High (2.85 + 0.15)
	MRSA-1354	Wound swab/cat	+	High (1.22 + 0.11)
	MRSA-1456	Wound swab/cat	+	High (1.50 + 0.22)
*S. pseudintermedius*	MRSP-149	Wound swab/dog	−	ND
	MSSP-517	Urine/dog	−	ND
	MSSP-527	Ear swab/dog	−	ND
	MSSP-438	Wound swab /dog	−	ND
	MSSP-513	Urine/dog	−	ND
Coagulase-negative *Staphylococci*	CNS-684	Wound swab/cat	−	ND
	CNS-676	Urine/cat	−	ND
	CNS-677	Urine/cat	−	ND
	CNS-683	Urine/cat	−	ND

The results “+” indicates phage-susceptible; “−” indicates phage-resistant; ND = Not determine.

### Genome features and annotation of phage vB_SauM_VL10

**Figure 2:**
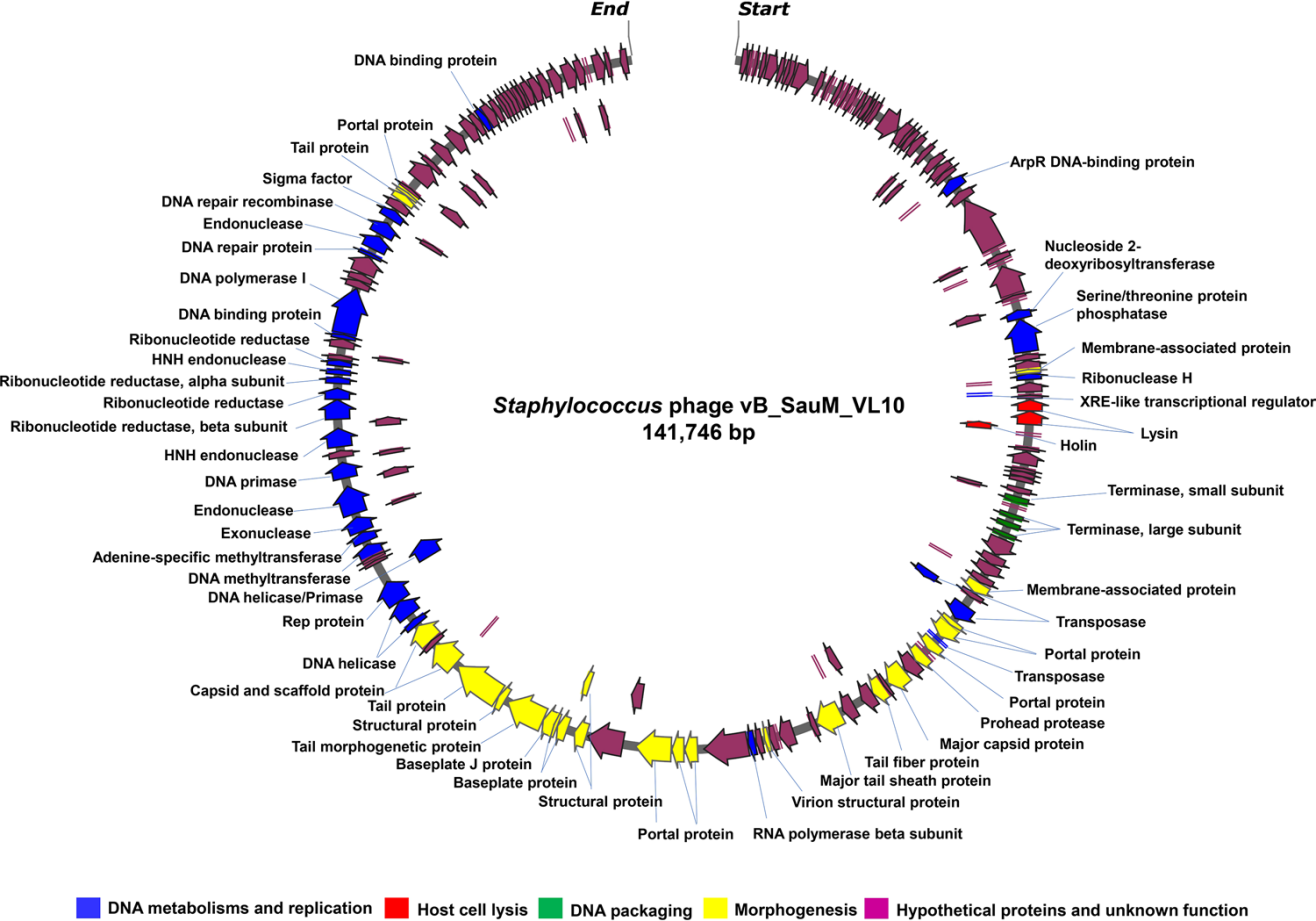
Schematic genome map of phage vB_SauM_VL10. A total of 193 open reading frames (ORFs) were annotated and indicated as arrows, where the arrowheads represented the orientation of the respective ORFs in the genome. The predicted function of each ORFs was marked with different colours including DNA metabolisms and replication (blue colour), host cell lysis (red colour), DNA packing (green colour), morphogenesis (yellow colour) and hypothetical protein and unknown function (purple colour).

Following genome sequencing and data processing, VL10 possesses a linear double-stranded DNA molecule with a length of 141,746 base pairs (bp) and an overall G + C content of 29.86%. The complete genomic sequence of VL10 has been deposited in the NCBI database under accession number OP940114. According to the PhageTerm^20^ analysis, the VL10 genome contains long direct terminal repeats (DTRs) of around 8422 bp, suggesting that the genetic material was packaged using the T5 phage (long DTRs) model^21^ (Supplementary data, Fig. S1). A total of 193 open reading frames (ORFs) were predicted as shown in Fig. 2. Among these, only 73 ORFs were assigned as putative of phage-related functional proteins. The predicted function of these ORFs were classified by functions into the following four categories that contain genes encoding for DNA metabolisms and replication, such as ribonuclease H (EC 3.1.26.4), RNA polymerase beta subunit, DNA helicase and primase, DNA methyltransferase, endonuclease (EC 2.1.1.72), HNH endonuclease, DNA polymerase I (EC 2.7.7.7), DNA-binding protein, XRE-like transcriptional regulator, transposase and DNA repair recombinase. Additional functional categories included host cell lysis such as lysin (EC 3.5.1.28) and holin and DNA packing with terminase small subunit and terminase large subunit. Gene involved in phage morphogenesis, including portal protein, major capsid protein, major tail sheath protein, baseplate, structural protein, tail fiber protein and capsid and scaffold protein. For more detailed information regarding the predicted ORFs and their functions, refer to supplementary data, Table S2. Remarkably, no genes encoding tRNA, bacterial virulence factors or antimicrobial resistance-related functions were identified in the phage genome.

Despite *in-silico* predictions by PhageAI suggesting a virulent phage lifestyle for VL10, up to 98.58%, we sought to validate the possibility of genome integration into host cells. After subjecting the bacterial cells to two rounds of gradually increasing multiplicity of infection (MOI), we observed that the surviving bacterial cells had become VL10-resistant *S. aureus* strains. This was evidenced by the failure of their chromosomal DNA to amplify using primers specific to the major capsid protein gene of VL10 (Supplementary data, Fig. S2). This finding demonstrates that VL10 is incapable of integrating its genome into *S. aureus* host cells, assuring its virulent nature. Consequently, this suggests that VL10 holds the potential for safe utilization in therapeutic applications.

### Genomic comparison and phylogenetic analysis of phage vB_SauM_VL10 with related phages

**Figure 3:**
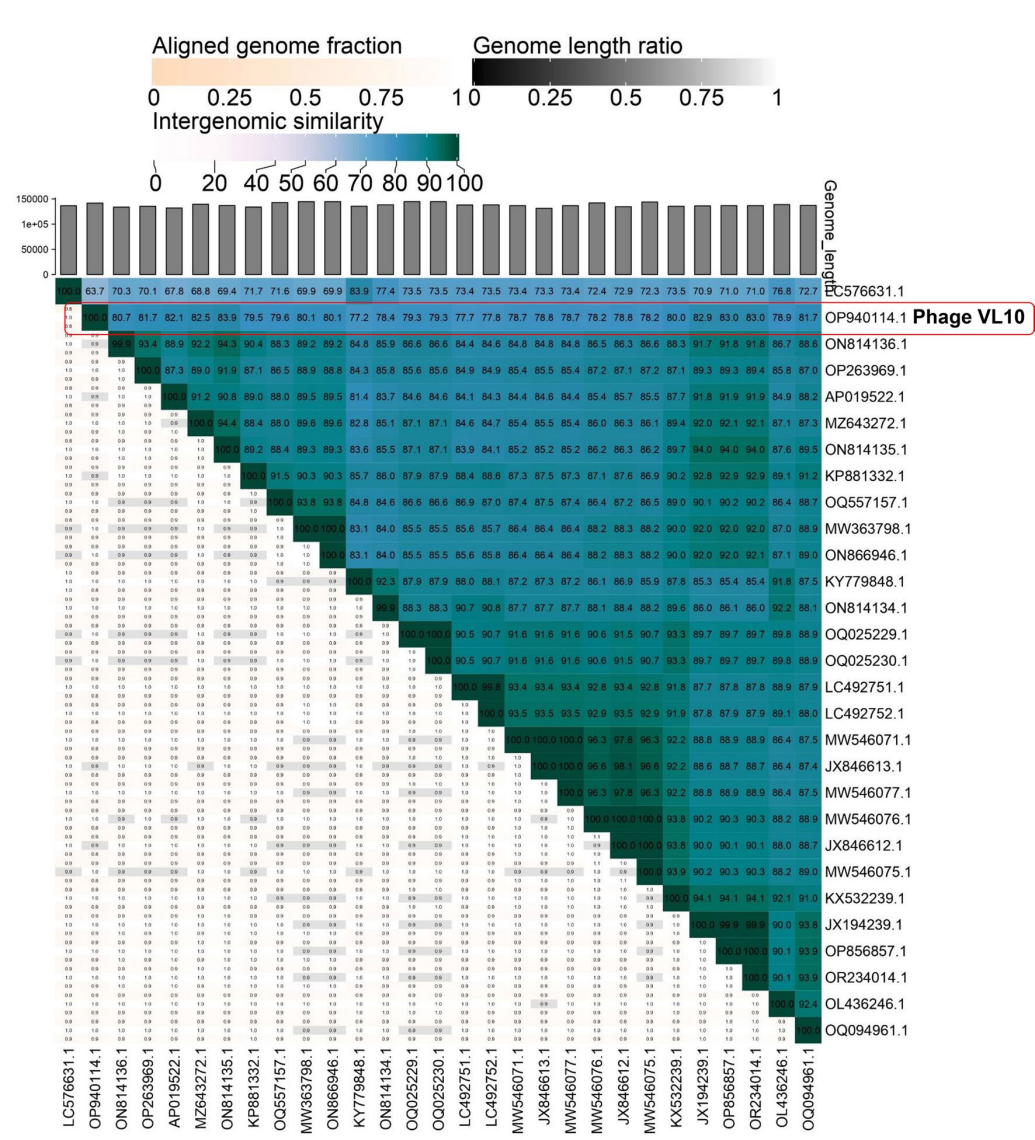
Heat map comparing complete genome of phage vB_SauM_VL10 to related *Staphylococcus* phages in the *Silviavirus* genus. The heat map was created with the VIRIDIC software (http://rhea.icbm.uni-oldenburg.de/VIRIDIC/), with the species differentiation threshold set to 95%. The percentage of homology is represented by the numbers in the chart.

**Figure 4:**
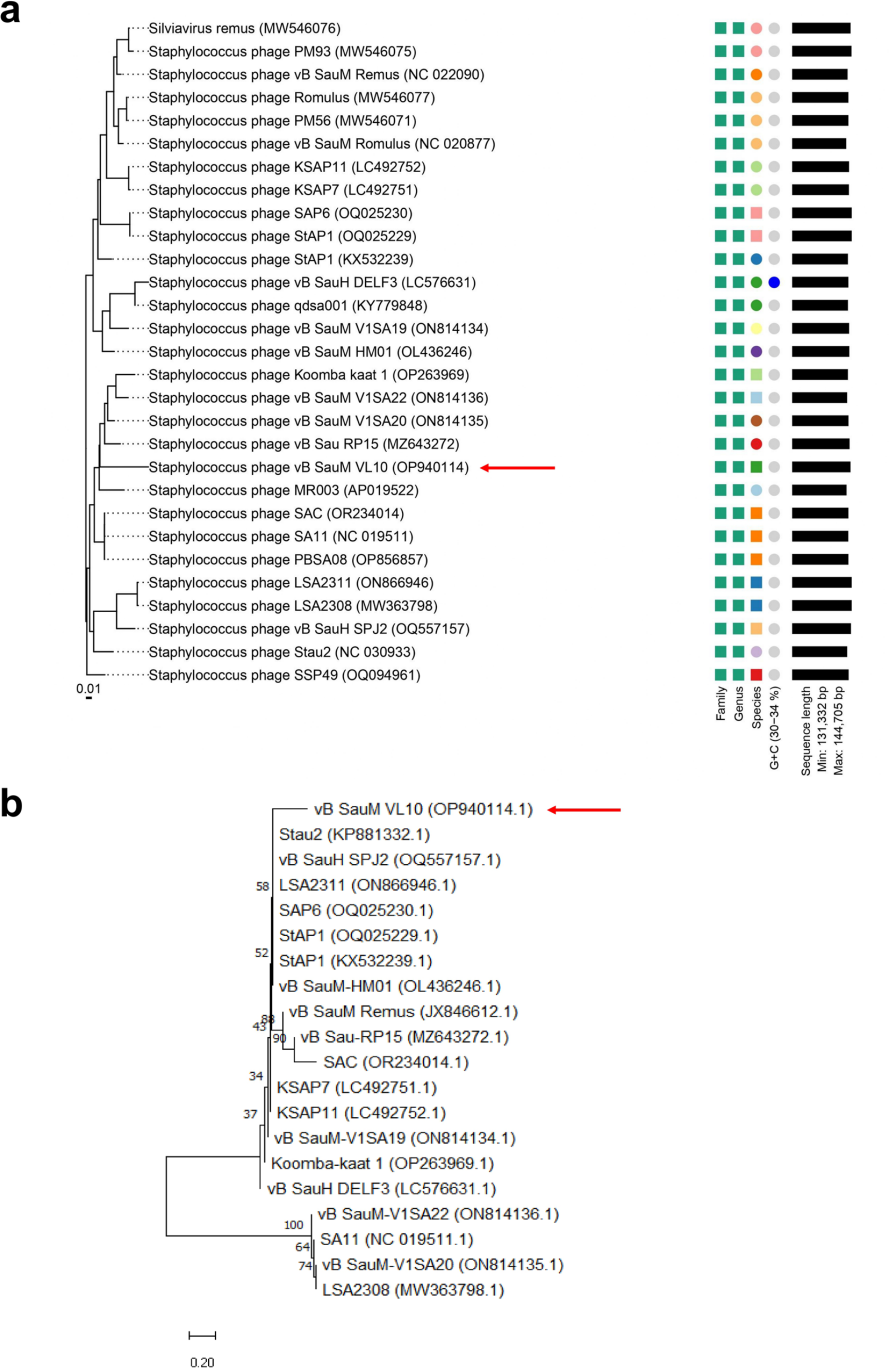
Phylogenetic analysis of phage vB_SauM_VL10 with other members of the *Silviavirus* genus (**a**) The Genome-BLAST Distance phylogenetic tree constructed using 29 whole genome sequences by VICTOR, an online program with settings suggested for phages (prokaryotic viruses). The study revealed phage VL10 to be a novel species in the *Silviavirus* genus*.* Neighbor-Joining phylogenetic tree based on (**b**) DNA polymerase gene of phage VL10 and related phages in *Silviavirus* genus. Numbers are displayed next to the branches indicating the percentage of replicate trees of the bootstrap test (1000 replicates). The Maximum Composite Likelihood was used to compute the evolutionary distances. Red arrows represent the phage VL10.

A Blastn (Megablast) comparison of the complete genome sequence of VL10 against the NCBI non-redundant database was conducted to classify the newly isolated phage at various taxonomic levels, including species, genus, subfamily and family. According to the recent taxonomy updated by the International Committee on Taxonomy of Viruses (ICTV)^22^, the analysis revealed that the VL10 genome shares a high degree of similarity with *Staphylococcus* phage genome sequences within the *Silviavirus* genus, subfamily *Twortvirinae*, family *Herelleviridae*, and class *Caudoviricetes*, with up to 97.93% nucleotide identity over 85% query coverage (Supplementary data, Table S3). To classify phage VL10 accurately, pairwise intergenomic similarities were calculated by the VIRIDIC program among various phage members of the *Silviavirus* genus. The result demonstrated that VL10 showed the highest nucleotide identity (83.9%) with *Staphylococcus* phage vB_SauM-V1SA20 (NCBI accession number: ON814135.1) and the lowest nucleotide identity (63.7%) with *Staphylococcus* phage vB_SauH_DELF3 (LC576631.1), as shown in Fig. 3 and Supplementary data, Table S4. Furthermore, a Genome-BLAST Distance Phylogeny (GBDP) tree generated using VICTOR identified phage VL10 as a separate species within the *Silviavirus* genus (Fig. 4a). In agreement with the criteria recommended by the International Committee on Taxonomy of Viruses (ICTV) for novel species identification, the homology of VL10 to other *Silviavirus* phages was found to be below the species threshold of 95%. Consequently, VL10 has been classified as a novel species within the *Silviavirus* genus.

To further investigate the evolutionary relationship between the VL10 and related phages in the *Silviavirus* genus, the DNA polymerase gene was selected for constructing a neighbor-joining (NJ) phylogenetic tree. For the DNA polymerase gene comparison, VL10 shared a common clade ancestor with the DNA polymerase of *Staphylococcus* phage Stau2^23^ (KP881332.1), which was isolated from a medical specimen in Taiwan, with a divergence of 0.27 base substitutions per site (Fig. 4b). The phylogenetic analysis confirms the status of VL10 as a novel species within the *Silviavirus* genus, emphasizing its unique evolutionary position among related phages.

### Characterisation of bacteriophage growth, lytic activity, and stability

**Figure 5:**
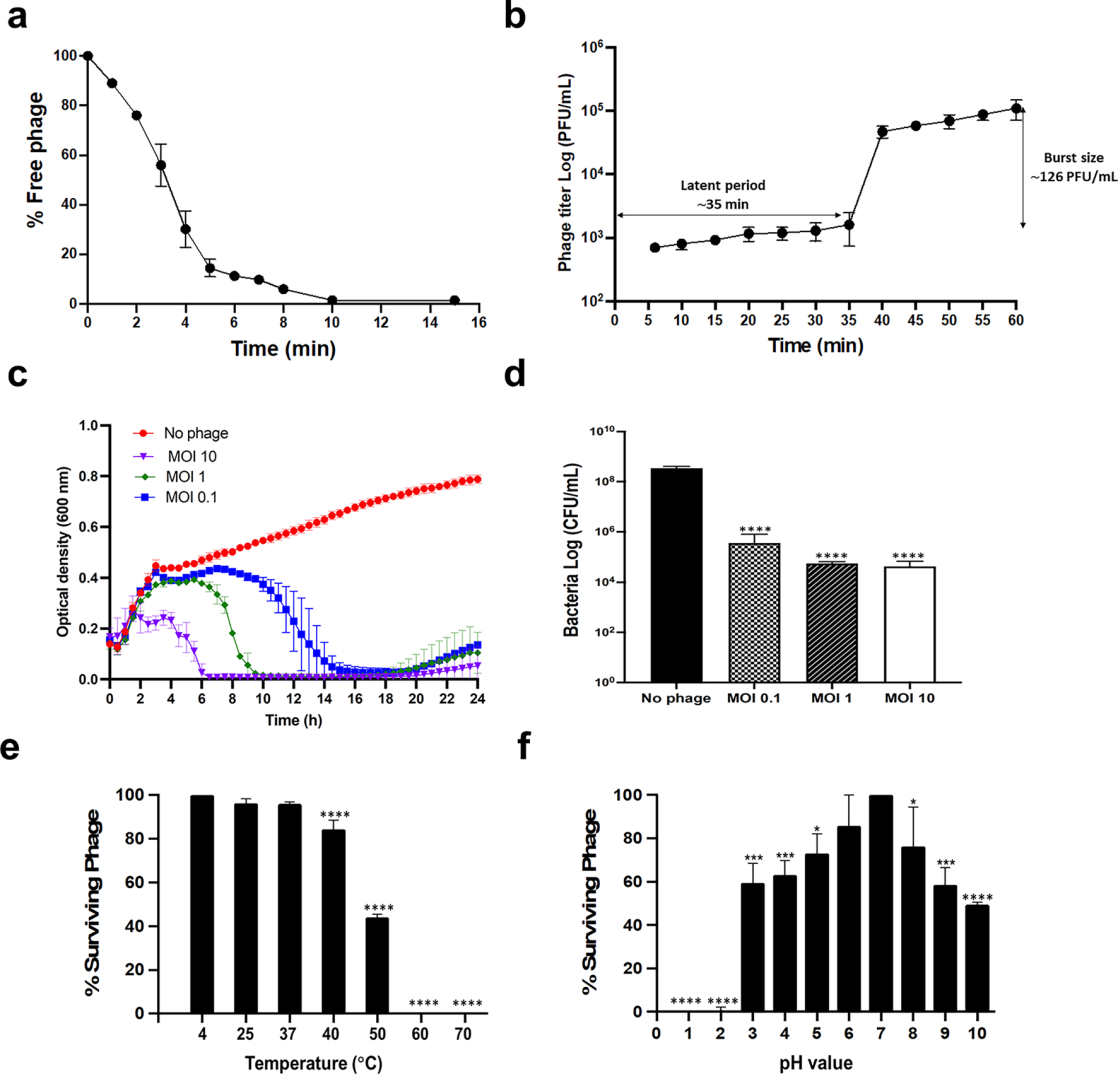
In vitro characterization of the phage vB_SauM_VL10 growth, bacterial killing activity and stability on the host strain (MRSA ATCC 43300). (**a**) Phage kinetic adsorption to the host strain surfaces at an MOI of 0.1. (**b**) One-step growth curve analysis of phage VL1 at a MOI of 0.1. (**c**) Bacterial killing activity of phage VL10 against host cells in LB medium. The OD_600_ was measured every 30 min for 24 h. (**d**) The impact of phage VL10 treatment on host cell viability, the host cells were cocultured with the phage at various MOIs and incubated at 37 °C for 24 h before counting viable bacterial cell counts (CFU/ml). Bacterial culture without phages was included as a control. (**e**) Stability of Phage VL10 under different temperature conditions. (**f**) Phage VL10 stability at different pH conditions. The data are presented as the mean ± standard deviation of three independent experiments. The asterisks indicate significant differences between the experimental and control groups (*P < 0.05, ***P < 0.001 or ****P < 0.0001, one-way ANOVA followed by Dunnett's post hoc test).

The experimental approach initiated with determining the optimal multiplicity of infection (MOI) for VL10, revealing that an MOI of 0.1 yielded the highest phage production (Supplementary data, Fig. S3). Consequently, for the construction of the adsorption and one-step growth kinetics assays, the rates were assessed at an MOI of 0.1. It was observed that over 90% of phage particles rapidly adsorbed to MRSA ATCC 43300 within 5 min at 37 °C (Fig. 5a). In terms of phage growth, the one-step growth curve analysis demonstrated that VL10 had a latent period of approximately 35 min, with each infected cell producing an average burst size of ~ 126 plaque-forming units (PFU) (Fig. 5b).

To evaluate the bacterial lytic activity of VL10, we monitored MRSA ATCC 43300 growth in broth culture medium during phage infection for 24 h at MOIs of 0.1, 1 and 10. The results showed that the optical density (OD600) of MRSA cultures without phage treatment (control) demonstrated continuous bacterial growth over 24 h. In contrast, under phage treatment conditions, the OD_600_ values of MRSA cultures at all MOIs (0.1, 1 and 10) only increase at the beginning of incubation, followed by a gradual decline until 20 h after infections. Subsequently, bacterial growth resumed but remained lower than that of the untreated control until the end of the experiment (Fig. 5c). Consistently, a significant reduction in cell viability was sustained throughout a 24-h period at all tested MOIs (*P* < 0.0001), resulting in a 3- to 4-log decrease in the number of colony-forming units (CFU) compared to the non-phage-treated control (Fig. 5d).

Furthermore, the stability of VL10 in various environmental conditions was assessed to determine optimal storage conditions. In terms of thermal stability, VL10 displayed high stable activity at 25 °C and 37 °C after 1 h of incubation, with phage titers comparable to the control at 4 °C. However, at higher temperatures of 40 °C and 50 °C, phage activity considerably declined (*P* < 0.0001). Phage activity was completely lost at temperatures exceeding 60 °C (Fig. 5e). Additionally, VL10 exhibited limited pH tolerance, remaining stable at pH levels between 6 and 7. The phage significantly reduced its infective activity in more acidic conditions (pH 3 to 5) and more alkaline conditions (pH 8 to 10) compared to pH 7 (*P* < 0.05). At extremely low pH values of 2 and 1, VL10 was completely inactive (Fig. 5f). These comprehensive characterisations demonstrate the efficacy, viability, and environmental stability of VL10, supporting its potential utility in phage therapy applications.

### Treatment of biofilm-formed MRSA by phage vB_SauM_VL10

**Figure 6:**
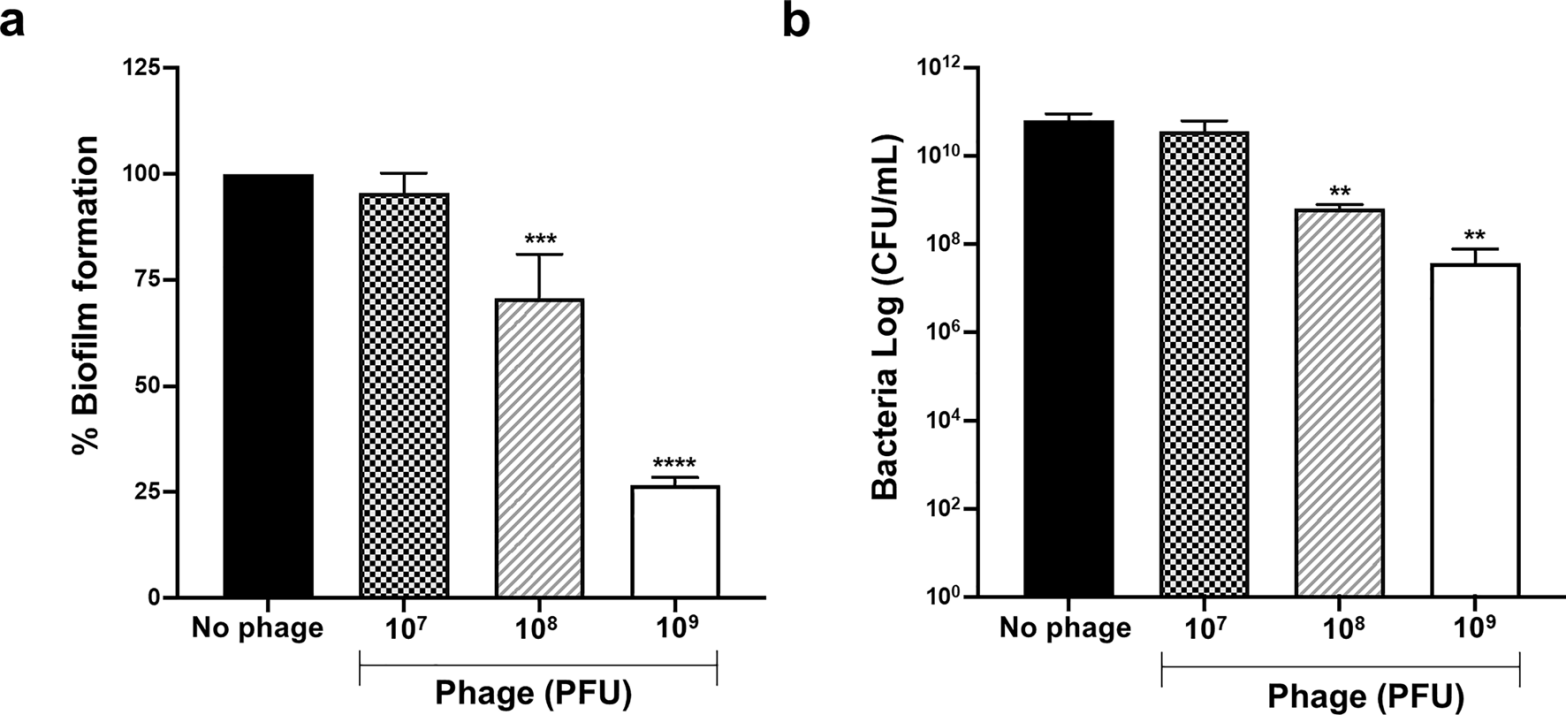
Biofilm eradication activity of the phage vB_SauM_VL10 against MRSA ATCC 43300 biofilm formed on polystyrene surfaces (**a**) The total biofilm mass of 48 h-old biofilms of MRSA ATCC 43300 treated with/without phage VL10 for 24 h. After a crystal violet stain, the absorbance of each well was measured at 595 nm. (**b**) Viable bacterial cell counts in 48 h-old biofilms of MRSA ATCC 43300 treated with/without phage VL10 for 24 h. were determined using the conventional plate count. The data are presented as the mean ± standard deviation of three independent experiments. The asterisks indicate significant differences between the experimental and control groups (**P < 0.01, ***P < 0.001 or ****P < 0.0001, one-way ANOVA followed by Dunnett's post hoc test).

MRSA often grows within biofilms, which are frequently associated with chronic and recurring infections^8,24^. To evaluate the biofilm-eradicating potential of VL10, MRSA biofilms were cultivated on polystyrene plates for 48 h, allowing them to mature. Subsequently, VL10 at various titers (10^7^, 10^8^ and 10^9^ PFU/mL) were introduced to the biofilms for 24 h. Crystal violet staining of the biofilm masses demonstrated that VL10, particularly at concentrations of 10^8^ and 10^9^ PFU/mL, significantly reduced established MRSA biofilms by 29.30% (*P* = 0.0007) and 73.27% (*P* < 0.0001), respectively, compared to the untreated control. However, treatment of the MRSA biofilms with VL10 at 10^7^ PFU/mL did not yield a significant reduction in biomass (Fig. 6a). This suggests that phage VL10 has potential as a biofilm-eradicating agent against MRSA biofilms, particularly at higher concentrations.

Furthermore, we evaluated the ability of VL10 to kill MRSA within biofilms. Treatment with phage concentrations of 10^8^ and 10^9^ PFU/mL resulted in a significant reduction in viable cells by 2-log (*P* = 0.0056) and 3.2-log (*P* = 0.0060), respectively. Conversely, treatment with VL10 at a concentration of 10^7^ PFU/mL did not yield a significant difference in the number of viable bacteria (Fig. 6b). These findings underscore the enhanced efficacy of higher concentrations of VL10 in targeting and killing MRSA within biofilms.

### Effectiveness of phage vB_SauM_VL10 in treating MRSA systemic infection in *G. mellonella* larvae

**Figure 7:**
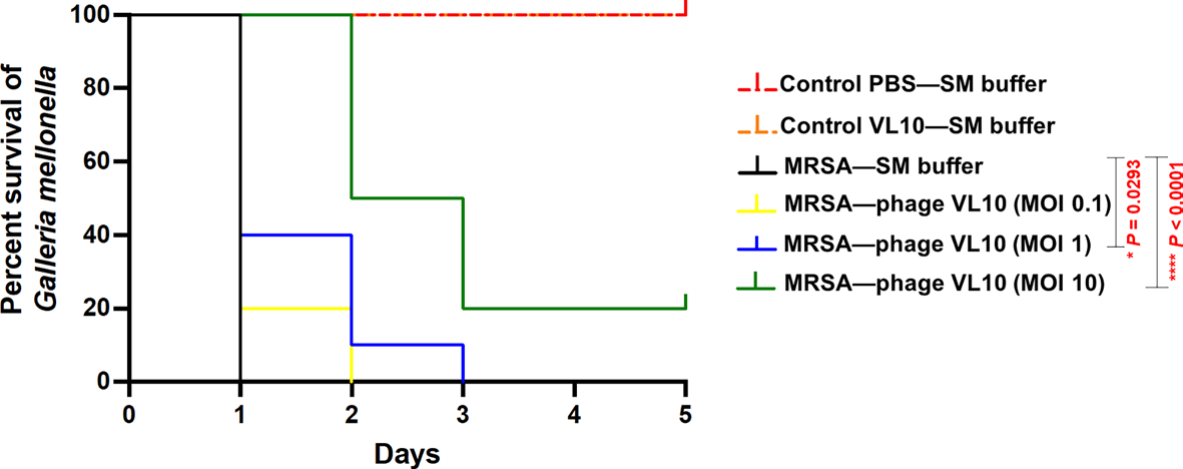
Percent survival of *G. mellonella* larvae infected with MRSA ATCC 43300 after treatment with phage vB_SauM_VL10. Groups of 10 larvae were infected with MRSA ATCC 43300 for an hour, followed by treatment with phage VL10 to achieve MOIs of 0.1, 1 or 10. Every day for 5 days post infection, the number of dead larvae was counted. The data was graphed and analysed using the GraphPad Prism software with a Log-rank (Mantel-Cox) test. Asterisks denoted statistically significant differences (*P < 0.05 or ****P < 0.0001) between the bacterial infection and phage treatment groups. Data is representative of that obtained in three independent experiments (n = 3 biological replicates).

To evaluate the in vivo therapeutic potential of VL10 for MRSA infection treatment, we employed a wax moth larvae (*G. mellonella*) model, using the same bacterial strain as in the in vitro experiments. Remarkably, no mortality was observed in *G. mellonella* larvae administered PBS, SM buffer, or phage lysate over a 5-day period, confirming the safety of phage VL10 as a therapeutic agent for larvae. In contrast, larvae infected with MRSA but not treated with phage VL10 succumbed to the infection within 1 day (Fig. 7).

For the treatment experiments, the in vivo efficacy of phage VL10 was assessed in MRSA-infected larvae at different phage doses represented by MOI of 0.1, 1 and 10. When compared to the group of MRSA-infected larvae without phage treatment, at MOI 0.1, no significant difference (*P* > 0.05) in the survival rate of MRSA-infected larvae was observed after treatment with VL10. However, with an increase in MOI to 1, VL10 significantly improved the survival rate to 20% and 10% at 1- and 2-days post infections, respectively and delayed the time to death in the MRSA-infected larvae (*P* = 0.0293). Furthermore, at MOI of 10, VL10 significantly increased the survival rate in MRSA-infected larvae to 20% (*P* < 0.0001) by the end of the 5-day observation period (Fig. 7). These results highlight the effectiveness of VL10 in enhancing the survival of MRSA-infected *G. mellonella* larvae.

### Phenotypic characterization of VL10-resistant *S. aureus* strains

**Figure 8:**
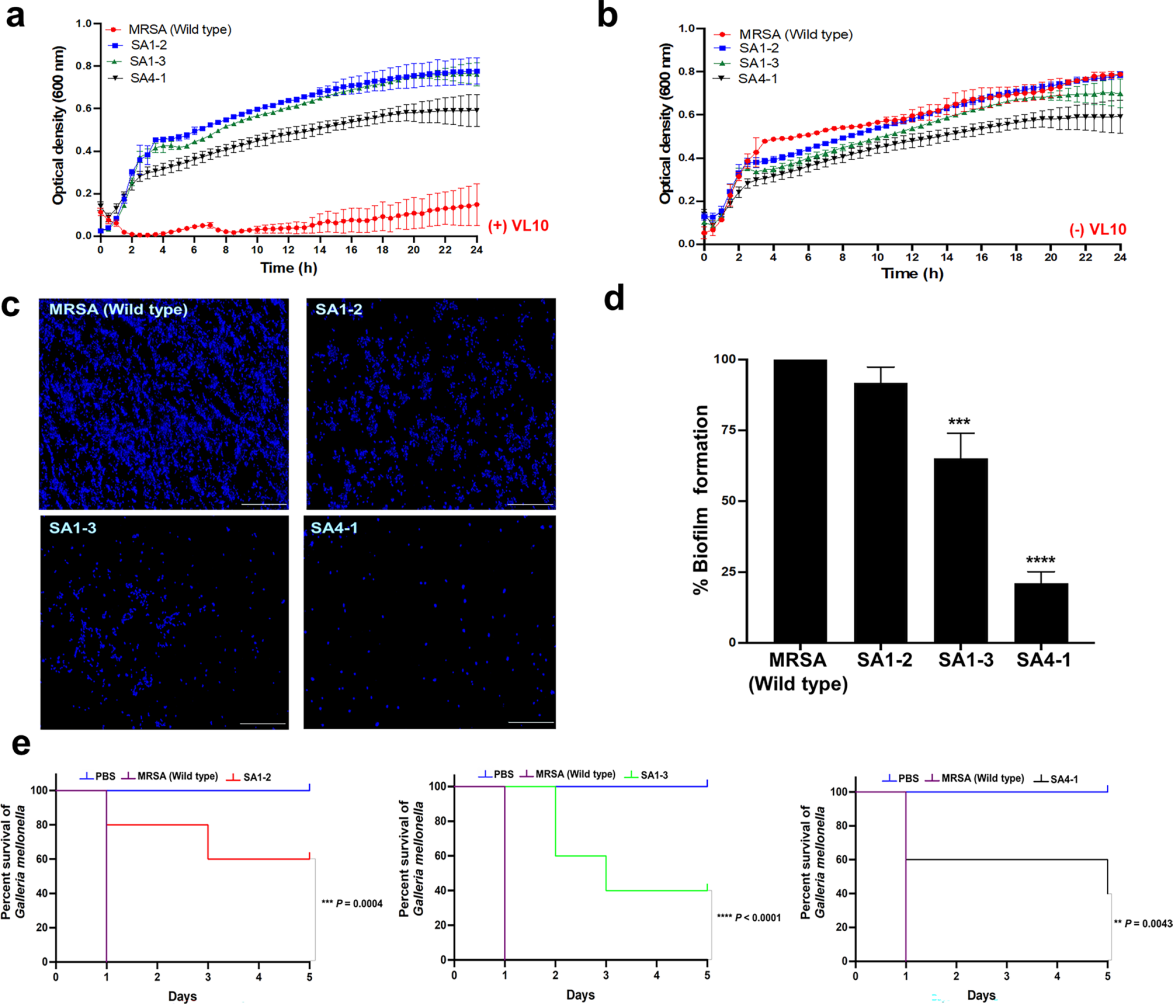
Comparison of biological properties between MRSA ATCC 43300 (wild type) and vB_SauM_VL10-resistant *S. aureus* strains (SA1-2, SA1-3 and SA4-1). (**a**,**b**) Bacterial growth curve of *S. aureus* strains treated with or without phage VL10 at MOI of 100. (**c**,**d**) The total biofilm mass assessed by a crystal violet staining and fluorescence microscope images of 48 h-old biofilms of *S. aureus* strains, stained with Hoechst 33342 (350/461 nm-blue). The scale bar represents 50 µm. Data presented as the mean ± standard deviation of three independent experiments. The asterisks indicate significant differences between the experimental and control groups (***P < 0.001 or ****P < 0.0001, one-way ANOVA followed by Dunnett's post hoc test). (**e**) Virulence of *S. aureus* strains in *G. mellonella* infection. Representative data from an experiment where groups of 10 larvae were infected with 10^7^ CFU of either *S. aureus* strains. The number of dead larvae was counted daily for 5 days post infection. Data analysed using the GraphPad Prism software with a Log-rank (Mantel-Cox) test. Data is representative of that obtained in three independent experiments (n = 3 biological replicates). Asterisks denoted statistically significant differences ***P < 0.001 or ****P < 0.0001) between the larvae infected with wild type and the VL10-resistant *S. aureus* strains.

Phage therapy, while effective in targeting bacteria, can lead to the development of phage-resistant mutants within bacterial populations. These mutants typically exhibit reduced overall fitness, diminished biofilm formation, and a decrease in virulence. This phenomenon underscores the complex interplay between phages and bacteria, highlighting the importance of ongoing research to optimize therapeutic outcomes^25–27^. Understanding these trade-offs is essential for optimizing phage therapy. Although the development of MRSA resistance to VL10 was not apparent during the initial 18 h of treatment, resistance became detectable after 24 h. VL10-resistant *S. aureus* strains, namely SA1-2, SA1-3, and SA4-1, were randomly selected as representatives for further investigation, with their confirmation achieved through PCR analysis (Supplementary data, Fig. S2). To evaluate the bacterial growth curve in the presence of VL10 at an MOI of 100, the growth curve of MRSA ATCC43300 (wild type) was effectively suppressed by VL10. However, the growth of phage-resistant mutant strains SA1-2, SA1-3, and SA4-1 remained unaffected by the phage, indicating its ineffectiveness in killing these mutants (Fig. 8a). Notably, despite the phage resistance exhibited by SA1-3 and SA4-1, a trade-off effect was seemingly observed in its growth properties, with SA1-3 and SA4-1 exhibiting slower growth compared to the wild type (Fig. 8b and Supplementary data, Fig. S2). When assessing biofilm formation using fluorescence microscopy, the 48-h-old MRSA (wild type) and SA1-2 biofilms displayed a dense structure on the polystyrene plates (Fig. 8c). In contrast, the bacterial density within biofilms formed by SA1-3 and SA4-1 visibly diminished. This observation was further supported by crystal violet staining of the biofilm masses, showing a significant decrease in biofilm mass in strains SA1-3 (34.8% reduction; *P* = 0.0002) and SA4-1 (79% reduction; *P* < 0.0001) compared to MRSA (wild type) (Fig. 8d). Additionally, the survival rate of *G. mellonella* larvae infected by phage-resistant mutant strains SA1-2, SA1-3, and SA4-1 was significantly higher than that of the wild-type strain (60%, 40%, and 40% vs 0%; *P* = 0.0004, *P* < 0.0001, and *P* = 0.0043, respectively) (Fig. 8e). This indicates that the phage-resistant mutants exhibited lower virulence. Altogether, our findings indicate that in developing resistance to VL10, certain bacteria undergo trade-offs in bacterial fitness. This trade-off manifests as defects in bacterial growth, reduced biofilm production, and attenuation in virulence.

## Discussion

*S. aureus*, a member of the ESKAPE group of antibiotic-resistant pathogens, poses a significant global health concern by evading conventional antibiotic treatments^28^. Hence, identifying alternative antimicrobial strategies is crucial, with phage therapy emerging as a compelling candidate. Therefore, this study aimed to isolate a *Staphylococcus* phage and thoroughly characterize its biology, genomics, and therapeutic potential. The findings provide valuable insights into the practical application of phage therapy against MRSA infections, emphasizing its importance as a promising alternative antimicrobial strategy.

Following established techniques utilized in previous studies^14,17,18,29^, we successfully isolated a novel *Staphylococcus* phage VL10, by screening in urban sewage samples for its activity against MRSA. The discovery of VL10 in urban sewage emphasizes the importance of exploring diverse environments for phage discovery. This finding is especially significant because sewage-contaminated environments serve as the principal reservoir for numerous multidrug-resistant bacterial strains and new phages^30,31^.

Whole genome sequencing of phages is crucial in understanding their genetic makeup, potential for fighting bacteria, and applications in phage therapy and biotechnology. The genome analysis of VL10 revealed its belonging to the *Silviavirus* genus, known for its effective anti-*S. aureus* phages with the broadest activity spectrum and display suitable properties for phage therapy application^13,14,17^. The gene products predicted for VL10 closely resemble those found in other phages within the *Silviavirus* genus^14,17,32^ and lacks genes for bacterial virulence or antibiotic resistance, making it a promising candidate for therapy. Additionally, within VL10 genome, ORFs58 and 59 encode lysins, which can break down peptidoglycan in bacterial cell walls, causing the bacterial cell to be lysed or destroyed^33^. Lysin of VL10 contains peptidoglycan recognition proteins, acting as pattern recognition receptors capable of binding to bacterial cell wall peptidoglycans and, in certain cases, catalysing the hydrolysis of bacterial cell wall peptidoglycans. While phages that target Gram-positive bacteria are typically host-specific, their lysins often exhibit a broad host spectrum^34^. Further research is needed to determine if this is the case for VL10. Phage VL10 genome also lacks genes related to integrating into host bacteria such as integrase enzymes. However, it encodes a DNA repair recombinase, likely playing an essential role in maintaining phage genome structure including processes like genome repair, circularization and replication^35^. There are at least six kinds of recombinase genes have been reported in phage genomes, two (UvsX and Gp2.5) found in virulent phages and four (Sak, Redβ, Erf and Sak4) in temperate phages^35^. Interestingly, the recombinase protein of VL10 exhibits a remarkable 98% identity (100% query cover) with the UvsX-like recombinase of lytic *Staphylococcus* phages^17,36,37^. Furthermore, VL10 encodes transposase proteins that, like those reported in other *Staphylococcus* lytic phages, are projected to exhibit splicing behaviour and create a functional portal protein^38^. Although an *in-silico* analysis indicated that VL10 is a virulent phage, experimental data demonstrated that it does not integrate into host cells, making it safe for therapeutic application. This safety aspect is crucial, as temperate phages can potentially transfer new genes to host bacteria^12,39^. Phylogenetic analysis and genomic comparisons placed VL10 in the *Silviavirus* genus. It was identified as a novel species within this genus, highlighting its uniqueness in the phage world. The analysis also revealed a close relationship with other phages isolated from different geographic locations (Supplementary data, Table S4), suggesting the potential widespread distribution of phages with comparable genetic features.

The biological properties of VL10 highlight its efficient adsorption to the MRSA host, with over 90% absorbed within 5 min. This rapid adsorption rate echoes the effectiveness observed in another member of the *Silviavirus* genus, specifically phage Stau2^23^. Such findings emphasize the significant efficacy of both phages in attaching to their respective host bacteria, which is a crucial step in initiating the infection process. Examining the growth parameters of VL10 reveals a remarkably shorter latent period of 35 min and a larger burst size of 126 PFU per infected cell. Comparing these characteristics to other members of the *Silviavirus* genus, VL10 exhibits a similar latent period to phage ΦMR003, which has a latency of 33 min. However, VL10 demonstrates a comparatively higher burst size than ΦMR003^17^, which typically yields an average burst size of 35 PFU per cell. Another *Silviavirus*, phage LSA2308^32^, exhibits a shorter latent period (20 min) and a larger burst size (467 PFU) than VL10. These findings underscore the unique growth dynamics of VL10, highlighting both its similarities and distinctions compared to other phages within the *Silviavirus* genus. Such properties indicative of a highly productive lytic cycle underscores the potential of phage as a bactericidal agent against pathogens, potentially reducing phage-resistant bacteria emergence^12^. Phages exhibiting excellent stability across a range of temperatures and pH levels are ideal choices for use as sanitizers or alternative therapeutic agents^40^. However, VL10 displayed its stability at 25 °C and 37 °C, as well as within a pH range of 6 to 7, making it suitable for storage and potential application under conditions similar to human blood (i.e., ∼37 °C and pH ∼7). The activity of VL10 was inactive when the temperature exceeded 50 °C or under extremely basic or acidic pH. These results align with a previous report that demonstrated how LSA2308 exhibited a rapid decrease in its activity when exposed to a temperature of 50 °C^32^. These conditions result in the denaturation of phage proteins and, subsequently, a loss of their viability^41^. Furthermore, VL10 exhibits a broad lytic spectrum and a high degree of target specificity, typical characteristics of *Silviavirus* members^13,14,17,23^. These characteristics are of exceptional value when phage dealing with mixed bacterial populations. Interestingly, VL10 effectively suppressed MRSA growth in a broth culture medium, with inhibition increasing as the MOI rose. A complete clearance of bacterial optical density occurred until 24 h post incubation, indicating effective bacterial lysis. This highlights VL10 potential in phage therapy for controlling MRSA populations, even at lower MOIs. A low phage dose in phage therapy provides safety, specificity, efficacy, and reduces resistance risk, making it the preferred choice for various therapeutic applications, including acute infections and scenarios needing lower phage concentrations^42^.

Biofilm-associated MRSA infections pose a major clinical challenge due to their resistance to antibiotics and tendency for chronic, recurrent infections^43^. The phage genomes can contain genes encoding biofilm matrix-degrading enzymes, including various hydrolytic enzymes. These enzymes have the potential to enhance the diffusion of phages within the biofilm matrix, facilitating their effectiveness^9,44^. The ability of VL10 to disrupt MRSA biofilms and a substantial reduction in viable MRSA within biofilms over the 24-h of treatment period highlights the potential of VL10 in combating biofilm-associated MRSA infections, which are well-recognized for their resistance to conventional antibiotics. Our findings align with previous studies that demonstrated the effectiveness of phages EW27, EW36, EW41, and EW71 in reducing MRSA biofilms formed by various clinical isolates^45^. Furthermore, research involving phage SDQ has shown its effectiveness in significantly reducing MRSA biofilms formed on various surfaces^18^. Another study evaluated the use of monophage Sb-1 and polyphage PYO, in preventing and eradicating MRSA biofilms. High phage concentrations effectively inhibited planktonic MRSA growth in a concentration-dependent manner, but complete MRSA biofilm eradication demanded prolonged co-incubation with the highest phage titers^10^.

Our study investigated the therapeutic potential of VL10 using a *G. mellonella* larvae model, which is a valuable and ethically acceptable model for studying bacterial infections in vivo^46^. Our findings demonstrate that VL10 significantly improves the survival rate of MRSA-infected larvae, suggesting its potential as a therapeutic agent against systemic MRSA infections. Comparing with previous studies, trends emerge: phages Sb-1 and PYO enhanced larval survival against MRSA infections^10^, while vB_SauM-A, vB_SauM-C, and vB_SauM-D increased survival rates and time against MDRSA strains^15^. Remarkably, phage therapy outperformed antibiotics in enhancing larval survival, highlighting its effectiveness in combating MRSA infections. These collective results underscore phage therapy’s promise as a superior approach to antibiotics in treating MRSA infections.

The emergence of phage-resistant mutants during phage therapy presents a significant challenge, highlighting the need to understand the associated fitness trade-offs for effective treatment strategies. In our study, phenotypic characterization of VL10-resistant *S. aureus* strains, revealed intriguing insights into their altered characteristics compared to the wild-type strain. Despite VL10’s inability to effectively kill these mutants, some bacterial mutants displayed notable weaknesses, including diminished overall bacterial fitness, reduced biofilm formation, and attenuated virulence. These observations highlight the intricate relationship between phage resistance and bacterial fitness, suggesting potential avenues for optimizing therapy. For instance, as bacteria develop phage resistance to phage and lose their ability to form biofilms, they may become more susceptible to elimination by antibiotics. Encouragingly, all resistant mutants in our study exhibited evident attenuation in virulence, suggesting potential advantages in managing corresponding infections. These attenuated mutants may be more susceptible to clearance by the host immune response, aiding in the resolution of infections. These characteristics are consistent with previous reports such as JJ01-resistant *Pseudomonas aeruginosa* strains, which incurred a bacterial fitness cost by producing less biofilm and exhibiting slower growth than parental strains, thereby increasing antibiotic susceptibility^27^. Additionally, phage-resistant *Klebsiella pneumoniae* strains isolated during phage P24 or P39 infection demonstrated decreased virulence in *G. mellonella* larvae, attributed to mutations in capsule polysaccharide genes and exopolysaccharide genes resulting in phage resistance^26^. Furthermore, whole-genome sequencing and analysis of SAVM01 and SAVM02-resistant mutants, isolated during *Staphylococcus* phage infection, identified several mutations on these mutant genomes, particularly in the *lexA* gene, responsible for regulating the response to DNA damage (SOS response)^47^ with additional mutations affecting various genes related to bacterial functions. These findings provide insights into the genetic basis of phage resistance mechanisms in *S. aureus*, shedding light on potential targets for future therapeutic strategies^48^. Therefore, further investigations involving whole-genome sequencing and analysis of VL10-resistant *S. aureus* strains will elucidate how bacteria trade physiological fitness for bacteriophage resistance, resulting in attenuated virulence.

In summary, VL10 holds promise as a therapeutic agent against MRSA infections, given its efficient antimicrobial activity, biofilm-disrupting ability, and in vivo efficacy. Further research is required to advance its clinical application.

## Materials and methods

### Ethics statement

All manipulations involving *S. aureus* were conducted within a Biosafety Level 2 (BSL2) facility according to standard operating procedures. The specific protocol for *S. aureus* and bacteriophage was approved by the Institutional Biosafety Committee-IBC, Faculty of Veterinary Science, Mahidol University. The protocol numbers are IBC/MUVS-B-004/2564 and IBC/MUVS-B-012/2566. All animal experiments were approved by the Faculty of Veterinary Science, Mahidol University Animal Care and Use Committee. The animal ethics approval certificate are FVS-MU-IACUC approval no. MUVS-2023-04-24, MUVS-2023-04-26 and MUVS-2023-06-39.

### Bacterial strains, growth conditions and antimicrobial susceptibility assay

The bacterial strains used in the present study are listed in Table 1. The four MRSA strains (DMST 20646, 20649, 20651 and 20652) were purchased from the Culture Collection for Medical Microorganisms, Department of Medical Sciences, Thailand. The thirty *S. aureus* clinical isolates were recovered from glycerol stocks of Department of Microbiology and Immunology, Faculty of Tropical Medicine, Mahidol University, and confirmed *S. aureus* character by conventional biochemical test (catalase, coagulase, DNase, glucose and mannitol fermentation). While six *S. aureus*, five *S. pseudintermedius* and four Coagulase-negative *Staphylococci* (CNS) strains were isolated from cat or dog clinical specimens as part of routine service at the Microbiology Laboratory, Center of Veterinary Diagnosis, Faculty of Veterinary Science, Mahidol University. All bacteria were grown in brain heart infusion broth (HiMedia) and Luria–Bertani broth (Invitrogen) with aeration at 37 °C in a shaking incubator at 150 rpm for 24 h. They were incubated inverted for 24 h at 37 °C in a solid medium.

The disk diffusion method on Muller Hinton agar (Oxoid™, UK) was performed to determine the antimicrobial susceptibility profile of *S. aureus* clinical isolates and the results were interpreted in accordance with the recommendations of the Clinical and Laboratory Standards Institute (CLSI) guidelines^49,50^. Antibiotic discs (Oxoid™) and concentrations were penicillin (10 Unit), erythromycin (15 μg), chloramphenicol (30 μg), trimethoprime/sulphamethoxazole (1.25 μg/23.75 μg), gentamicin (10 μg), ciprofloxacin (5 μg), levofloxacin (5 μg), norfloxacin (10 μg), tetracycline (30 μg), rifampin (5 μg), clindamycin (2 μg), doxycycline (30 μg). Cefoxitin (30 μg) and oxacillin (1 μg) discs were used for phenotypic identification of methicillin-resistant isolates. The reference strain, *S. aureus* ATCC25923, 29213 and 43300 were acquired from the American Type Culture Collection (ATCC) and used as the quality control for MSSA or MRSA, respectively.

### Phage isolation, purification, and preparation

Phage was isolated from wastewater collected from several locations in Bangkok, Thailand using *S. aureus* ATCC25923 as the host strain for phage isolation and propagation. Briefly, 100 mL of the raw sewage samples were filtrated through 0.22 µM pore-size membrane filters (Millipore, USA) to remove the bacterial debris and then mixed with an equal amount of double-strength LB broth (HiMedia, India) containing the early log phase (1%) of *S. aureus* ATCC25923 for phage enrichment. The culture was cultured at 37 °C and shaken at 150 rpm overnight before being harvested and filtered using a 0.22 µM membrane filters. To detect phage plaque, 10 µL of supernatant was spotted on the *S. aureus* ATCC25923 lawn and overnight incubated at 37 °C. To purify the isolated phage, an individual plaque was picked and passaged at least five times by a double-layer plaque assay technique. To prepare the high-titer stock for downstream experiment, the purified phage VL10 was propagate in *S. aureus* ATCC25923, following the previously described method^29^.

### Transmission electron microscopy analysis

To examine virion morphology, a drop of filtrated phage suspension (10^9^ PFU/mL) was applied into a Formvar-carbon-coated copper grid (200 or 300 mesh) and negatively stained with 2% uranyl acetate for 10 min followed by several washes. The stained grids were subsequently air-dried on Whatman No. 1 filter paper and examined using a transmission electron microscope (FEI, TECNAI T20) with an acceleration voltage of 120 kV.

### Optimal multiplicity of infection

The multiplicity of infection (MOI) is defined as the ratio of the phage to the host bacteria during the processes of infection. To determine the optimal MOI of phage as previously described^18,51^, the mid-log phase of MRSA ATCC 43300 (approximately 10^8^ CFU/mL) were co-culture with phage VL10 suspensions at different MOIs (0.1, 1, 10 and 100) for 8 h at 37 °C with shaking at 180 rpm. After centrifugation (12,000 rpm, 5 min), phage titers of the supernatant were measured using a double-layer plaque assay. Three independent experiments were performed.

### Adsorption and one-step growth kinetics assays

The adsorption efficiency of phage to adsorb to the host surface (MRSA ATCC 43300) was determined as previously described^52^ with some modifications. Briefly, the mid-log phase of the MRSA ATCC 43300 suspension was infected with the phage in fresh LB medium containing 2 mM CaCl_2_ at an MOI of 0.1. The mixture was immediately aliquoted into separate vials followed by incubation at 37 °C. At various post-infection time intervals (0, 1, 2, 3, 4, 5, 6, 7, 8, 10 and 15 min), the mixture was filtered through a 0.45 μm pore size filter, and the titers of unabsorbed phages were determined using a double-layer plaque assay.

The phage latent period and average burst size were assessed by conducting a one-step growth kinetics analysis as previously described^53^ with some adjustments. Briefly, MRSA ATCC 43,300 was infected with a phage at an MOI of 0.1 in LB medium supplemented with 2 mM CaCl_2_. Based on the adsorption curve (Fig. 5a), the one-step growth curve was outlined by incubating the VL10 with the host MRSA ATCC 43300 for 5 min at 37 °C with shaking at 150 rpm to facilitate adsorption of the phage to the bacterial cells. After adsorption, the mixture was rapidly centrifuged (13,000 rpm, 45 s) to remove unadsorbed phage and resuspended in a fresh prewarmed medium. Subsequently, it was incubated at 37 °C with shaking at 150 rpm. The phage titer was measured immediately after this step, defined as time 6 post-infection (T6). At 10, 15, 20, 25, 30, 35, 40, 45, 50, 55, and 60 min post-infection, phage titers were measured using a double-layer plaque assay. Countable plaques were plotted to generate curves used to determine the phage latent period and burst size, following previously described methods. All experiments were conducted in triplicate.

### Bacterial killing assay, host range determination and efficiency of plating (EOP)

The killing activity of phage VL10 on the bacterial host was assessed by infecting the mid-log phase of MRSA ATCC 43300 (approximately 10^8^ CFU/mL) with phage suspensions at MOIs of 0.1, 1, 10, and 100 in a 48-well microtiter plate containing LB medium. The bacterial growth was monitored by measuring the absorbance at OD_600_ using a microplate reader (Molecular Devices, USA) every 30 min for 24 h. Bacterial cultures without phages served as control samples. In addition, after 24 h post-infection, the number of viable MRSA ATCC 43300 cells at various MOIs was determined by a colony counting assay. Results are presented as the average of three replicates.

The host range of phage VL10 was determined by a standard spot test and verified using a double-layer plaque assay. In brief, 10 μL of phage suspension (10^8^ PFU/mL) was dropped across a lawn of tested bacterial strains (Table 1). The production of a clear lysis zone after an overnight incubation at 37 °C was recorded as (+) = lytic (phage-susceptible) or (−) = non-lytic (phage-resistant). Any bacterial host cells lytic results were confirmed using double-layer plaque assays and further assessed the efficiency of plating (EOP). The EOP value was calculated by dividing the number of plaques on each susceptible bacterial strain by the number of plaques obtained from the original host strain (MRSA ATCC43300). Following the computation, EOP is divided into four categories; high (EOP ≥ 0.5), moderate (EOP > 0.1– < 0.5), low (EOP ≤ 0.1) and no activity (EOP < 0.001).

### Thermal and pH stability assays

The effects of temperature on phage stability were studied at 4, 25, 37, 40, 50, 60 and 70 °C. The phage suspension (10^8^ PFU/mL) in SM buffer pH 7.4 was incubated for an hour at various temperatures. After all treatments, the double-layer technique was then used to assess the phage titers. The relative titer was calculated as the ratio of phage titers at a different temperature to those stored at 4 °C. To test the influence of different pH values on phage stability, 10^8^ PFU/mL of phage VL10 was incubated individually for an hour at 37 °C in SM buffer ranging from pH 1 to 10 before phage titer counting. The relative titer was computed as the ratio of phage titers treated with different pH levels to those of the original SM buffer (pH 7.4). Three independent experiments were performed.

### Lysogeny verification assay

To investigate the potential for phage VL10 to integrate its genome into the bacterial genome and form a lysogenic cycle, a lysogeny test was conducted as previously described^54^ with minor modification. The bacterial host MRSA ATCC43300 in LB broth was infected with phage VL10 at MOI of 0.01 and incubated at 37 °C with shaking at 150 rpm for 24 h. The infected culture was then diluted 1:100 in LB broth and phage VL10 was added at an MOI of 10^4^ and one hour of incubation. Subsequently, 100 μL of the culture was plated and incubated at 37 °C for 48 h. The single bacterial colonies were picked and isolated in pure cultures following tested for phage-resistant strains by spot test. The potential for phage VL10 to integrate its genome into the bacterial genome and form a lysogenic cycle was confirmed by PCR. The genomic DNA of the obtained phage-resistant strains serve as a template for PCR-amplification using primers specific to the phage VL10 major capsid protein gene (VL10cap-F 5ʹCAGGTGCTTTA CGTCGTGAA3ʹ and VL10cap-R 5ʹGACGCTACTCCGATTTCACG3ʹ). For the positive control of bacterial DNA, primers (341f, and 785r) for 16s rRNA gene as previously described^55^ were used.

### Biofilm eradication assay

The biofilm-eradicating activity of phage VL10 was determined according to the previous method^18,56^ with minor modification. To establish biofilms, MRSA ATCC43300 was diluted to a final concentration of 10^8^ CFU/mL in TSB medium containing 1% d-glucose and incubated in 24-well flat-bottomed polystyrene microplates (Corning, USA) at 37 °C for 48 h. After incubation, the established biofilms were rinsed with phosphate-buffered saline (PBS; Sigma, USA) and phage VL10 solution (approximately 10^7^, 10^8^ or 10^9^ PFU/mL) was added to each well and incubated at 37 °C for 24 h. Subsequently, the total biofilm mass was assessed using crystal violet staining and the absorbance was measured at OD_595_ in the microplate spectrophotometer. Established biofilms without phage treatment were used as controls. Additionally, the number of viable bacterial cells within biofilms was enumerated by serially diluting the contents of each well in PBS before plating on LB plates. The experiments were performed in triplicates. To determine biofilm formation of phage-resistant mutants in comparison to MRSA ATCC43300 (Wild type), 10^8^ CFU/mL of VL10-resistant mutant strains (SA1-2, SA1-3 and SA4-1) or MRSA ATCC43300 (Wild type) were grown in TSB medium containing 1% D-glucose and incubated in 24-well flat-bottomed polystyrene microplates at 37 °C for 48 h. Subsequently, the total biofilm mass was assessed as described.

The microscopic imaging of MRSA biofilms was conducted following a previously established protocol^18^. Initially, bacterial biofilms were cultivated on glass coverslips and subsequently rinsed twice with sterile PBS to eliminate non-adherent cells. Following this, the biofilms underwent direct staining with Hoechst 33342, according to the manufacturer’s protocol, before being visualised under fluorescence microscopy (Leica DMi8, Leica Microsystems, Germany).

### Phage therapy assay in a *G. mellonella* infection model

The therapeutic effect of phage VL10 was assessed in an in vivo test using a *G. mellonella* larvae model based on a previous method with some alterations^10^. Larvae weighing approximately 300 mg with a cream color and without signs of weakness were selected for experiments. The larvae were separated into four groups, each containing 10 larvae. In the treatment group, larvae were injected with 10 µL of MRSA ATCC43300 suspension containing 10^7^ CFU into the last left-side proleg. One hour after the injection of the bacteria, 10 µl of a phage VL10 suspension at a concentration of 10^6^, 10^7^ and 10^8^ PFU (MOI of 0.1, 1 and 10, respectively) were inoculated via the last right-side proleg. The larvae in the non-treatment group were infected with the same dosage as the treatment group but treated with SM buffer. Two negative control groups were included, with one group consisting of larvae injected with PBS and treated with SM buffer and the other group consisting of larvae injected with a phage suspension and treated with SM buffer. After treatment, the larvae were maintained at a temperature of 37 °C within a dark and humid environment. Their survival was assessed daily for a period of 5 days. To assess the virulence of phage-resistant mutants in comparison to MRSA ATCC43300 (Wild type), larvae were injected with 10 µL of VL10-resistant mutant strains (SA1-2, SA1-3 and SA4-1) or MRSA ATCC43300 (Wild type) suspension containing 10^7^ CFU into the last proleg. Larvae injected with PBS alone served as negative controls. The larvae were then incubated at 37 °C, and the number of deceased larvae was recorded daily for up to 5 days. Larvae were considered dead if they displayed a darkened appearance and remained motionless when physically stimulated. This study was conducted through three separate and independent experiments.

### Genome sequencing and bioinformatic analysis

Phage genomic DNA was isolated from purified phage VL10 particles using the phenol/chloroform/isoamyl alcohol extraction method as follows^56^. The DNA sample was sent to Macrogen Inc., (Seoul, South Korea) for whole genome sequencing based on the Illumina sequencing platform. The initial raw reads were trimmed to remove adapter sequences. Following that, the filtered high-quality reads were used for de novo genome assembly, which was accomplished using SPAdes^57^ (version 3.13.0).

The complete genome sequence of the phage VL10 was analysed using rapid annotation using the subsystem technology (RAST)^58^ platform. Open reading frames (ORFs), were predicted, and ORF functions were annotated using the protein basic local alignment search tool (Blastp). Protein motif predictions were conducted using the Conserved Domain Search Service (CD search) of the NCBI database. A genome map of phage VL10 was constructed and visualized using the SnapGene tool trial version 2023. The termini of the phage genome and the packing mechanism were in silico predicted using PhageTerm^20^. Putative tRNAs in the genome were identified using tRNA Scan-SE^59^. Antimicrobial resistance (AMR) genes and variants were predicted using the Resistance Gene Identifier (RGI) tool incorporated in the Comprehensive Antibiotic Resistance Database (CARD) server^60^. Potential virulence factors encoded by phages were examined by BLASTP algorithm against a virulence factor database (VFDB)^61^. The phage lifecycle was predicted online by PhageAI^62^. Pairwise intergenomic similarities between phage VL10 and the related phage genomes were calculated using the VIRIDIC tool^63^.

### Construction of phylogenetic tree

To assess the phylogenetic relationship between the complete genome nucleotide sequences of phage VL10 and other phage members of the *Silviavirus* genus, the VICTOR web service (https://ggdc.dsmz.de/victor.php) was used to generate a GBDP tree. In order to analyse the genetic relationships by using DNA polymerase genes of the phage in *Silviavirus* genus were aligned using the MAFFT online server^64^. Following, the neighbor-joining phylogenetic trees were constructed by the MEGA software version 11 and bootstrap percentage analyses were based on 1000 replications.

### Statistical analysis

The data are shown as the mean and standard deviation of three separate experiments. The GraphPad Prism program, version 9.2.0 (USA), was used to statistically analyze the data using the appropriate test. For multi-group comparisons, the mean of results was compared using one-way analysis of variance (ANOVA) followed by Dunnett's post hoc test. For survival curves, the Mantel-Cox log-rank test was applied. The significance level for differences between the compared experiments was set to less than 0.05.

### Supplementary Information

Supplementary Information.

## Data Availability

The complete genomic sequence of phage VL10 has been deposited in the Genbank database under accession number OP940114.1. Data is publicly available now.

## References

[1] Magill SS (2014). Multistate point-prevalence survey of health care-associated infections. N. Engl. J. Med..

[2] Tong SY, Davis JS, Eichenberger E, Holland TL, Fowler VG (2015). *Staphylococcus aureus* infections: Epidemiology, pathophysiology, clinical manifestations, and management. Clin. Microbiol. Rev..

[3] Kobayashi SD, Malachowa N, DeLeo FR (2015). Pathogenesis of *Staphylococcus aureus* abscesses. Am. J. Pathol..

[4] Kondo S (2022). Molecular characterization of methicillin-resistant *Staphylococcus aureus* genotype ST764-SCCmec type II in Thailand. Sci. Rep..

[5] Foster TJ (2017). Antibiotic resistance in *Staphylococcus aureus*. Current status and future prospects. FEMS Microbiol. Rev..

[6] Tacconelli E (2018). Discovery, research, and development of new antibiotics: The WHO priority list of antibiotic-resistant bacteria and tuberculosis. Lancet Infect. Dis..

[7] Sanchez CJ (2013). Biofilm formation by clinical isolates and the implications in chronic infections. BMC Infect. Dis..

[8] Peng Q, Tang X, Dong W, Sun N, Yuan W (2022). A review of biofilm formation of *Staphylococcus aureus* and its regulation mechanism. Antibiotics.

[9] Atshan SS (2023). Phage therapy as an alternative treatment modality for resistant *Staphylococcus aureus* infections. Antibiotics.

[10] Tkhilaishvili T, Wang L, Tavanti A, Trampuz A, Di Luca M (2020). Antibacterial efficacy of two commercially available bacteriophage formulations, *Staphylococcal* bacteriophage and PYO bacteriophage, against methicillin-resistant *Staphylococcus aureus*: Prevention and eradication of biofilm formation and control of a systemic infection of *Galleria mellonella* larvae. Front. Microbiol..

[11] Gorsi A (2018). Phage therapy: What have we learned?. Viruses.

[12] Fernandez L, Gutierrez D, Garcia P, Rodriguez A (2019). The perfect bacteriophage for therapeutic applications-a quick guide. Antibiotics.

[13] Saez Moreno D (2021). epsilon (2)-Phages are naturally bred and have a vastly improved host range in *Staphylococcus aureus* over wild type phages. Pharmaceuticals.

[14] Kolenda C (2022). Phage therapy against *Staphylococcus aureus*: Selection and optimization of production protocols of novel broad-spectrum *Silviavirus* phages. Pharmaceutics.

[15] Kazmierczak N, Grygorcewicz B, Roszak M, Bochentyn B, Piechowicz L (2022). Comparative assessment of bacteriophage and antibiotic activity against multidrug-resistant *Staphylococcus aureus* biofilms. Int. J. Mol. Sci..

[16] Fujiki J (2022). Biological properties of *Staphylococcus* virus PhiSA012 for phage therapy. Sci. Rep..

[17] Peng C (2019). *Silviavirus* phage ɸMR003 displays a broad host range against methicillin-resistant *Staphylococcus aureus* of human origin. Appl. Microbiol. Biotechnol..

[18] Song J (2021). Potential of bacteriophages as disinfectants to control of *Staphylococcus aureus* biofilms. BMC Microbiol..

[19] Nagel T (2022). Phage banks as potential tools to rapidly and cost-effectively manage antimicrobial resistance in the developing world. Curr. Opin. Virol..

[20] Garneau JR, Depardieu F, Fortier LC, Bikard D, Monot M (2017). PhageTerm: A tool for fast and accurate determination of phage termini and packaging mechanism using next-generation sequencing data. Sci. Rep..

[21] Wang J (2005). Complete genome sequence of bacteriophage T5. Virology.

[22] Turner D (2023). Abolishment of morphology-based taxa and change to binomial species names: 2022 taxonomy update of the ICTV bacterial viruses subcommittee. Arch. Virol..

[23] Hsieh SE, Lo HH, Chen ST, Lee MC, Tseng YH (2011). Wide host range and strong lytic activity of *Staphylococcus aureus* lytic phage Stau2. Appl. Environ. Microbiol..

[24] Proctor RA (2006). Small colony variants: A pathogenic form of bacteria that facilitates persistent and recurrent infections. Nat. Rev. Microbiol..

[25] Li N (2022). Characterization of phage resistance and their impacts on bacterial fitness in *Pseudomonas aeruginosa*. Microbiol. Spectr..

[26] Fang Q, Feng Y, McNally A, Zong Z (2022). Characterization of phage resistance and phages capable of intestinal decolonization of carbapenem-resistant *Klebsiella pneumoniae* in mice. Commun. Biol..

[27] Wannasrichan W (2022). Phage-resistant *Pseudomonas aeruginosa* against a novel lytic phage JJ01 exhibits hypersensitivity to colistin and reduces biofilm production. Front. Microbiol..

[28] Pendleton JN, Gorman SP, Gilmore BF (2013). Clinical relevance of the ESKAPE pathogens. Expert Rev. Anti Infect. Ther..

[29] Lubowska N (2019). Characterization of the three new *Kayviruses* and their lytic activity against multidrug-resistant *Staphylococcus aureus*. Microorganisms.

[30] Sib E (2019). Antibiotic resistant bacteria and resistance genes in biofilms in clinical wastewater networks. Int. J. Hyg. Environ. Health.

[31] Tan CS (2020). Could bacteriophages isolated from the sewage be the solution to methicillin-resistant *Staphylococcus aureus*?. Med. J. Malaysia.

[32] Ma F (2021). Bacteriophages LSA2308 and LSA2366 infecting drug-resistant *Staphylococcus aureus*: Isolation, characterization and potential application for milk safety. LWT-Food Sci. Technol..

[33] Fischetti VA, Nelson D, Schuch R (2006). Reinventing phage therapy: Are the parts greater than the sum?. Nat. Biotechnol..

[34] Zhang B (2022). Characterization and genomic analysis of a novel jumbo bacteriophage vB_StaM_SA1 infecting *Staphylococcus aureus* with two lysins. Front. Microbiol..

[35] Lopes A, Amarir-Bouhram J, Faure G, Petit MA, Guerois R (2010). Detection of novel recombinases in bacteriophage genomes unveils Rad52, Rad51 and Gp2.5 remote homologs. Nucleic Acids Res..

[36] Kim MS, Myung H (2012). Complete genome of *Staphylococcus aureus* phage SA11. J. Virol..

[37] Iszatt JJ (2022). Genome sequences of two lytic *Staphylococcus aureus* bacteriophages isolated from wastewater. Microbiol. Resour. Announc..

[38] Vandersteegen K (2013). Romulus and Remus, two phage isolates representing a distinct clade within the *Twortlikevirus* genus, display suitable properties for phage therapy applications. J. Virol..

[39] Ling H (2022). Recent advances in bacteriophage-based therapeutics: Insight into the post-antibiotic era. Acta Pharm. Sin. B.

[40] Fister S (2016). Influence of environmental factors on Phage-bacteria interaction and on the efficacy and infectivity of phage p100. Front. Microbiol..

[41] Hazem A (2002). Effects of temperatures, pH-values, ultra-violet light, ethanol and chloroform on the growth of isolated thermophilic *Bacillus* phages. New Microbiol..

[42] Loc-Carrillo C, Abedon ST (2011). Pros and cons of phage therapy. Bacteriophage.

[43] Lebeaux D, Ghigo JM, Beloin C (2014). Biofilm-related infections: Bridging the gap between clinical management and fundamental aspects of recalcitrance toward antibiotics. Microbiol. Mol. Biol. Rev..

[44] Azeredo J, Sutherland IW (2008). The use of phages for the removal of infectious biofilms. Curr. Pharm. Biotechnol..

[45] Whittard E (2021). Phenotypic and genotypic characterization of novel polyvalent bacteriophages with potent in vitro activity against an international collection of genetically diverse *Staphylococcus aureus*. Front. Cell Infect. Microbiol..

[46] Menard G, Rouillon A, Cattoir V, Donnio PY (2021). *Galleria mellonella* as a suitable model of bacterial infection: Past, present and future. Front Cell Infect. Microbiol..

[47] Podlesek Z, Zgur Bertok D (2020). The DNA damage inducible sos response is a key player in the generation of bacterial persister cells and population wide tolerance. Front. Microbiol..

[48] Plumet L (2023). Isolation and characterization of new bacteriophages against *Staphylococcal* clinical isolates from diabetic foot ulcers. Viruses.

[49] Humphries R, Bobenchik AM, Hindler JA, Schuetz AN (2021). Overview of changes to the clinical and laboratory standards institute performance standards for antimicrobial susceptibility testing, M100, 31st edition. J. Clin. Microbiol..

[50] CLSI. Performance standards for antimicrobial disk and dilution susceptibility tests for bacteria isolated from animals. 6th ed. CLSI supplement VET01S. (Clinical and Laboratory Standards Institute, 2023).

[51] Ji Y (2019). Preventive effect of the phage VB-SavM-JYL01 on rabbit necrotizing pneumonia caused by *Staphylococcus aureus*. Vet. Microbiol..

[52] Kropinski AM (2009). Measurement of the rate of attachment of bacteriophage to cells. Methods Mol. Biol..

[53] Kropinski AM (2018). Practical advice on the one-step growth curve. Methods Mol. Biol..

[54] D'Andrea MM (2017). phiBO1E, a newly discovered lytic bacteriophage targeting carbapenemase-producing *Klebsiella pneumoniae* of the pandemic Clonal Group 258 clade II lineage. Sci. Rep..

[55] Thijs S (2017). Comparative evaluation of four bacteria-specific primer pairs for 16S rRNA gene surveys. Front. Microbiol..

[56] Kim SG (2021). Two novel bacteriophages control multidrug- and methicillin-resistant *Staphylococcus pseudintermedius* biofilm. Front. Med..

[57] Bankevich A (2012). SPAdes: A new genome assembly algorithm and its applications to single-cell sequencing. J. Comput. Biol..

[58] Aziz RK (2008). The RAST server: Rapid annotations using subsystems technology. BMC Genomics.

[59] Chan PP, Lowe TM (2019). tRNAscan-SE: Searching for tRNA genes in genomic sequences. Methods Mol. Biol..

[60] Alcock BP (2020). CARD 2020: Antibiotic resistome surveillance with the comprehensive antibiotic resistance database. Nucleic Acids Res..

[61] Chen L, Zheng D, Liu B, Yang J, Jin Q (2016). VFDB 2016: Hierarchical and refined dataset for big data analysis–10 years on. Nucleic Acids Res..

[62] Tynecki P (2020). PhageAI - bacteriophage life cycle recognition with machine learning and natural language processing. BiorXiv..

[63] Moraru C, Varsani A, Kropinski AM (2020). VIRIDIC—A novel tool to calculate the intergenomic similarities of prokaryote-infecting viruses. Viruses.

[64] Katoh K, Rozewicki J, Yamada KD (2019). MAFFT online service: Multiple sequence alignment, interactive sequence choice and visualization. Brief Bioinform..

