# Supplementary material for: Isolation and characterisation of a novel Silviavirus bacteriophage promising antimicrobial agent against methicillin-resistant Staphylococcus aureus infections
Source: Sci Rep. 2024 Apr 22;14:9251. doi: 10.1038/s41598-024-59903-w (PMC11035597; doi:10.1038/s41598-024-59903-w)
Supplement: Supplementary file 1 — Supplementary Information. [file 41598_2024_59903_MOESM1_ESM.pdf]

Supplementary information

Isolation and characterisation of a novel *Silviavirus* bacteriophage promising antimicrobial agent against Methicillin-Resistant *Staphylococcus aureus* infections

Varintip Lerdsittikul[1✉], Sukanya Apiratwarrasakul[1], Thassanant Atithep[2], Patoo Withatanung[3], Nitaya Indrawattana[4,5], Pornpan Pumirat[4], Somjit Chaiwattanarungruengpaisan[6], & Metawee Thongdee[6]

[1]Veterinary Diagnostic Center, Faculty of Veterinary Science, Mahidol University, Nakhon Pathom, Thailand. [2]Frontier Research Center, Vidyasirimedhi Institute of Science and Technology, Rayong, Thailand. [3]Department of Immunology, Faculty of Medicine Siriraj Hospital, Mahidol University, Bangkok, Thailand. [4]Department of Microbiology and Immunology, Faculty of Tropical Medicine, Mahidol University, Bangkok, Thailand. [5]Siriraj center of research and excellence in allergy and immunology (SiALL), Faculty of Medicine Siriraj Hospital, Mahidol University, Bangkok, Thailand. [6]The Monitoring Surveillance Center for Zoonotic Diseases in Wildlife and Exotic Animals, Faculty of Veterinary Science, Mahidol University, Nakhon Pathom, Thailand.

✉Correspondence and requests for materials should be addressed to VL

(E-mail: Varintip.sri@mahidol.edu)

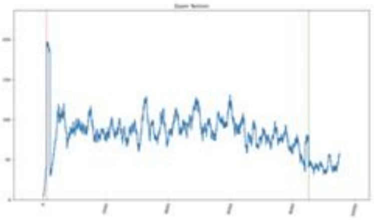

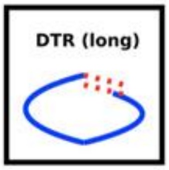

PhageTerm Method

Ends	Left (red)	Right (green)	Permuted	Orientation	Class	Type
Redundant	108	8529	No	NA	DTR (long)	T5

**Direct Terminal Repeats: 8422 bp*

Li's Method

Packaging	Termini	Forward	Reverse	Orientation
COS	Fixed	Obvious Termini	Obvious Termini	Forward

**Direct Terminal Repeats: 8422 bp*

Strand	Location	SPC	R
+	108	154	31
	5255	5	-
	1457	5	-
	5280	4	-
	5204	4	-
-	8529	38	8
	8160	5	-
	14047	4	-
	10067	4	-
	7464	4	-

SPC

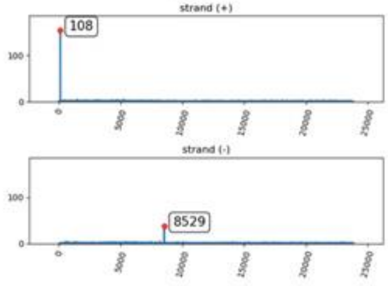

Figure S1. PhageTerm analysis of *Staphylococcus* phage vB_SauM_VL10 genome

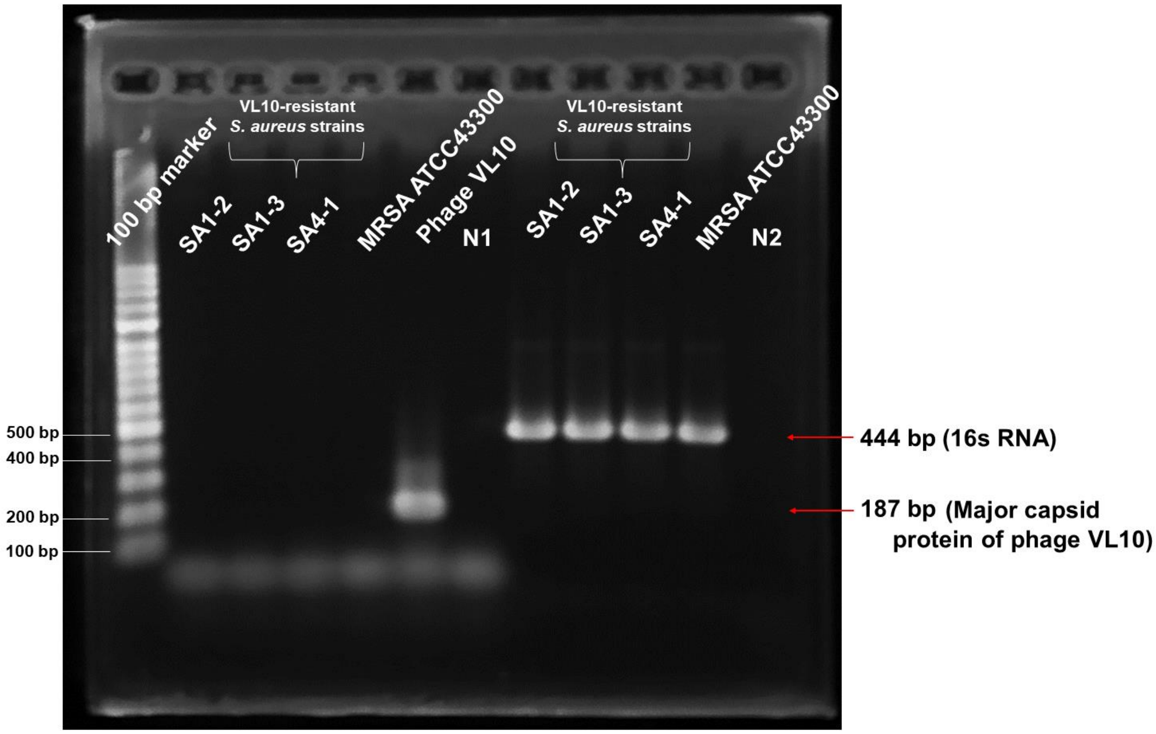

Figure S2. PCR amplification to confirm the inability of *Staphylococcus* phage vB_SauM_VL10 to integrate its genome into MRSA ATCC 43300 Host Cells. To assess the potential integration of phage VL10 genome into the host cells, a lysogeny test followed by PCR experiments was conducted. Genomic DNA extracted from VL10-resistant *S. aureus* strains (SA1-2, SA1-3 and SA4-1) was used as templates for amplification, using a pair of phage VL10-specific primers (targeting the major capsid protein gene, resulting in a 187 bp PCR product) and universal 16S rRNA bacterial primers (generating a 444 bp PCR product). Notably, all tested phage-resistant isolates did not yield a positive band for phage VL10-specific primers, but they exhibited a positive band for bacterial cells. As a positive control, phage VL10 DNA was included for phage-specific primer amplification. MRSA ATCC 43300 genomic DNA was employed as a negative control for phage-specific primers and as a positive control for bacterial primers. N1 and N2 represent the negative controls for the PCR experiments.

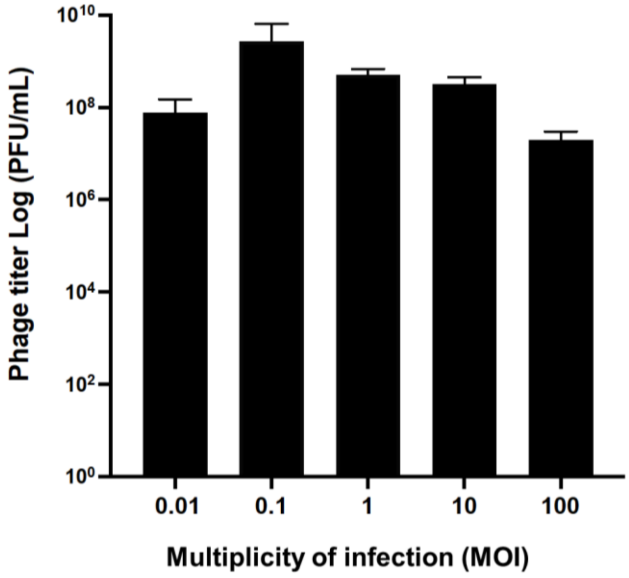

Figure S3. Determination of optimal multiplicity of infection (MOI) of *Staphylococcus* phage vB_SauM_VL10 in MRSA ATCC 43300 host cells. The values represent the means and standard deviations (SD) from three independent experiments (n = 3).

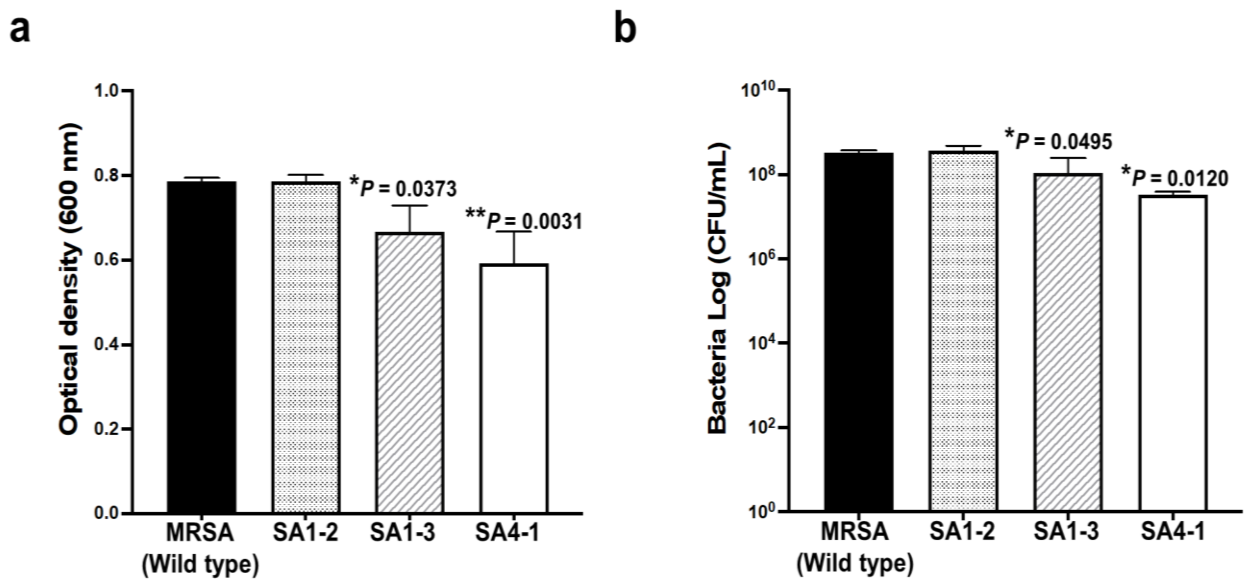

Figure S4. Bacterial growth of *S. aureus* strains, including MRSA ATCC 43300 (wild type) and vB_SauM_VL10-resistant *S. aureus* strains (SA1-2, SA1-3 and SA4-1), incubated at 37°C for 24 h. The growth was measured either by optical density at 600 nm (OD600) **(a)** or by counting viable bacterial cell counts (CFU/ml) **(b)**. The data are presented as the mean ± standard deviation of three independent experiments. The asterisks indicate significant differences between the experimental and control groups (*$P < 0.05$ or **$P < 0.01$, one-way ANOVA followed by Dunnett's post hoc test).

Table S1 Antibiotic susceptibility pattern of *Staphylococcus aureus* and *S. pseudintermedius* standard laboratory strains or isolates from different clinical samples. ND = Not determined

**Strains**	**PG**	**OX**	**FOX**	**E**	**CN**	**CIP**	**LEV**	**NOR**	**SXT**	**TE**	**RD**	**DA**	**DO**	**C**
ATCC 43300	ND	R	R	R	S	ND	ND	S	S	ND	ND	R	ND	ND
DMST 20646	ND	R	R	R	R	ND	ND	R	R	R	ND	R	ND	ND
DMST 20649	ND	R	R	R	R	R	ND	R	R	R	ND	R	ND	ND
DMST 20651	ND	R	R	R	R	R	ND	R	R	R	ND	R	ND	ND
DMST 20652	ND	R	R	R	R	R	ND	R	R	R	ND	R	ND	ND
MSSA-TM1	R	R	S	R	R	R	R	R	S	S	S	R	S	S
MRSA-TM2	R	R	R	R	R	R	R	R	S	S	S	R	S	S
MSSA-TM3	S	S	S	R	R	R	R	R	S	S	S	R	S	S
MSSA-TM4	S	S	S	S	S	S	S	S	S	S	S	S	S	S
MSSA-TM5	S	S	S	I	R	R	R	R	R	S	R	S	S	S
MSSA-TM6	R	S	S	I	I	S	S	S	S	S	S	S	S	S
MSSA-TM7	S	S	S	S	S	S	S	S	S	S	S	S	S	S
MSSA-TM8	S	S	S	S	S	S	S	S	S	S	S	S	S	S
MSSA-TM9	S	S	S	S	S	S	S	S	S	S	S	S	S	S
MSSA-TM10	S	S	S	S	S	S	S	S	R	S	S	S	S	S
MSSA-TM11	R	S	S	S	S	S	S	S	S	S	S	S	S	S
MSSA-TM12	R	S	S	S	S	S	S	S	R	S	S	S	S	S
MSSA-TM13	S	S	S	S	S	S	S	S	S	R	S	S	S	S
MSSA-TM14	S	S	S	S	S	S	S	S	S	R	S	S	R	S

Strains	PG	OX	FOX	E	CN	CIP	LEV	NOR	SXT	TE	RD	DA	DO	C
MSSA-TM15	R	S	S	S	S	R	I	R	S	S	S	S	S	S
MSSA-TM16	S	S	S	S	S	S	S	S	S	S	S	S	S	S
MSSA-TM17	S	S	S	S	S	S	S	S	S	S	S	S	S	S
MSSA-TM18	R	S	S	S	S	S	S	S	S	S	S	S	S	S
MSSA-TM19	R	R	S	S	S	S	S	S	S	S	S	S	S	S
MSSA-TM20	R	S	S	S	S	S	S	S	S	S	S	S	S	S
MSSA-TM21	R	S	S	S	S	S	S	S	S	S	S	S	S	S
MSSA-TM22	R	S	S	S	S	S	S	S	S	S	S	S	S	S
MSSA-TM23	R	S	S	S	S	S	S	S	R	S	S	S	S	S
MSSA-TM24	R	S	S	S	S	S	S	S	S	S	S	S	S	S
MSSA-TM25	S	S	S	S	S	S	S	S	S	S	S	S	S	S
MSSA-TM26	S	S	S	S	S	S	S	S	S	S	S	S	S	S
MSSA-TM27	S	S	S	S	S	S	S	S	S	R	S	S	S	S
MSSA-TM28	R	S	S	S	S	S	S	S	S	S	S	S	S	S
MSSA-TM29	S	S	S	S	S	S	S	S	S	S	S	S	S	S
MSSA-TM30	S	S	S	S	S	S	S	S	S	S	S	S	S	S
MSSA-279	ND	S	S	S	S	ND	ND	S	S	S	ND	S	S	S
MSSA-556	ND	S	S	S	S	ND	ND	S	S	S	ND	I	S	S
MRSA-461	ND	R	R	R	R	ND	ND	S	S	R	ND	S	R	S
MSSA-891	ND	S	S	S	S	ND	ND	S	S	S	ND	S	S	S
MRSA-1354	ND	R	R	S	R	ND	ND	R	R	ND	ND	R	ND	S

Strains	PG	OX	FOX	E	CN	CIP	LEV	NOR	SXT	TE	RD	DA	DO	C
MRSA-1456	ND	R	R	R	R	ND	ND	R	I	ND	ND	R	ND	ND
MRSP-149	ND	R	R	R	R	ND	ND	R	S	R	ND	S	R	S
MSSP-517	ND	S	S	S	R	ND	ND	R	R	R	ND	S	R	S
MSSP-527	ND	S	S	R	R	ND	ND	S	S	S	ND	R	S	S
MSSP-438	ND	S	S	S	R	ND	ND	S	S	S	ND	S	S	S
MSSP-513	ND	S	S	S	S	ND	ND	S	S	S	ND	S	S	S

PG=Penicillin (10 Unit), OX=Oxacillin (1 µg), FOX=Cefoxitin (30 µg), E=Erythromycin (15 µg), CN= Gentamicin (10 µg), CIP=Ciprofloxacin (5 µg), LEV= Levofloxacin (5 µg), NOR= Norfloxacin (10 µg), SXT= trimethoprime/sulphamethoxazole (1.25 µg/23.75 µg), TE=Tetracycline (30 µg), RD= Rifampin (5 µg), DA= Clindamycin (2 µg), DA=Doxycycline (30 µg) and C=Chloramphenicol (30 µg)

Table S2. General features of predicted open reading frame (ORF) in *Staphylococcus* phage vB_SauM_VL10 genome and homology to protein in NCBI database.

ORF/ Region	Coordinate		Strand	Length		Putative functions	Best BLASTp match and identification	Identity (%)	Conserved protein domain family (Name and accession)
	Start	Stop		bp	aa				
1	434	841	+	408	135	Hypothetical protein	Hypothetical protein [*Staphylococcus* phage vB_SauM-V1SA20]	94	
2	875	1105	+	231	76	Hypothetical protein	Hypothetical protein O151_gp067 [*Staphylococcus* phage vB_SauM_Remus]	69	
3	1106	1294	+	189	62	Hypothetical protein	Hypothetical protein SPJ221_5 [*Staphylococcus* phage vB_SauH_SPJ2]	69	
4	1312	1521	+	210	69	Hypothetical protein	Hypothetical protein O151_gp064 [*Staphylococcus* phage vB_SauM_Remus]	63	
5	1775	2083	+	309	102	Hypothetical protein	Hypothetical protein [*Staphylococcus* phage vB_SauM-HM01]	61	
6	2200	2946	+	747	248	Hypothetical protein	Hypothetical protein [*Staphylococcus* phage vB_SauM-V1SA22]	83	
7	3033	3356	+	324	107	Pentapeptide repeat protein	Pentapeptide repeat protein [*Staphylococcus* phage vB_Sau-RP15]	82	

ORF/ Region	Coordinate		Strand	Length		Putative functions	Best BLASTp match and identification	Identity (%)	Conserved protein domain family (Name and accession)
	Start	Stop		bp	aa				
8	3484	3765	+	282	93	Hypothetical protein	Hypothetical protein [*Staphylococcus* phage vB_SauM-HM01]	88	
9	3795	4097	+	303	100	Hypothetical protein	Hypothetical protein [Staphylococcus phage StAP1]	98	
10	4206	5153	+	948	315	Hypothetical protein	ORF030 [*Staphylococcus* phage Twort]	65	
11	5797	6207	+	411	136	Hypothetical protein	Hypothetical protein [*Staphylococcus* phage vB_SauM-V1SA22]	88	
12	6716	6504	-	213	70	Hypothetical protein	Hypothetical protein MRS_019 [*Staphylococcus* phage MR003]	97	
13	6918	6751	-	168	55	Hypothetical protein	Hypothetical protein OZ71_gp014 [*Staphylococcus* phage MCE-2014]	71	
14	7206	7520	+	315	104	Hypothetical protein	Hypothetical protein F422_gp027 [*Staphylococcus* phage SA11]	96	
15	7593	7748	+	156	51	Hypothetical protein	hypothetical protein [*Staphylococcus* phage vB_SauM-V1SA19]	94	
16	7750	7983	+	234	77	Hypothetical protein	Hypothetical protein [*Staphylococcus* phage vB_SauM-V1SA19]	93	

ORF/ Region	Coordinate		Strand	Length		Putative functions	Best BLASTp match and identification	Identity (%)	Conserved protein domain family (Name and accession)
	Start	Stop		bp	aa				
17	8065	8211	+	147	48	Hypothetical protein	Hypothetical protein [*Staphylococcus* phage vB_SauM-V1SA20]	88	
18	8264	8650	+	387	128	Hypothetical protein	No significant similarity found		
19	8664	8810	+	147	48	Hypothetical protein	Hypothetical protein SAP1_170 [*Staphylococcus* phage StAP1]	94	
20	8977	9267	+	291	96	Hypothetical protein	Hypothetical protein RP15_gp135 [*Staphylococcus* phage vB_Sau-RP15]	97	
21	9407	9748	+	342	113	Hypothetical protein	Hypothetical protein LSA2308_00003 [*Staphylococcus* phage LSA2308]	97	
22	9762	9983	+	222	73	Hypothetical protein	Hypothetical protein [*Staphylococcus* phage vB_SauM-V1SA19]	97	
23	10091	10435	+	345	114	Hypothetical protein	Hypothetical protein BH792_gp143 [*Staphylococcus* phage Stau2]	90	
24	10915	12195	+	1281	426	Hypothetical protein	Hypothetical protein OZ71_gp015 [*Staphylococcus* phage MCE-2014]	76	
25	12832	12266	-	567	188	Hypothetical protein	Hypothetical protein [*Staphylococcus* phage vB_SauM-V1SA19]	90	

ORF/ Region	Coordinate		Strand	Length		Putative functions	Best BLASTp match and identification	Identity (%)	Conserved protein domain family (Name and accession)
	Start	Stop		bp	aa				
26	13090	12851	-	240	79	Hypothetical protein	Hypothetical protein RP15_gp140 [*Staphylococcus* phage vB_Sau-RP15]	84	
27	13491	13093	-	399	132	Hypothetical protein	Hypothetical protein [*Staphylococcus* phage vB_SauM-V1SA19]	86	
28	14072	13551	-	522	173	Hypothetical protein	Hypothetical protein RP15_gp142 [*Staphylococcus* phage vB_Sau-RP15]	91	
29	14455	14072	-	384	127	Hypothetical protein	Hypothetical protein RP15_gp143 [*Staphylococcus* phage vB_Sau-RP15]	89	
30	14850	14458	-	393	130	Hypothetical protein	Hypothetical protein RP15_gp144 [*Staphylococcus* phage vB_Sau-RP15]	93	
31	15248	14853	-	396	131	Hypothetical protein	Hypothetical protein [*Staphylococcus* phage vB_SauM-V1SA22]	95	Yox super family (cl09859)
32	15799	15245	-	555	184	Hypothetical protein	Hypothetical protein Romulus_150 [*Staphylococcus* phage vB_SauM_Romulus]	84	PHA02241 super family (cl10405)
33	16485	15925	-	561	186	Hypothetical protein	Hypothetical protein RP15_gp149 [*Staphylococcus* phage vB_Sau-RP15]	98	

ORF/ Region	Coordinate		Strand	Length		Putative functions	Best BLASTp match and identification	Identity (%)	Conserved protein domain family (Name and accession)
	Start	Stop		bp	aa				
34	16894	16502	-	393	130	Hypothetical protein	Hypothetical protein RP15_gp150 [*Staphylococcus* phage vB_Sau-RP15]	96	
35	17142	16915	-	228	75	Hypothetical protein	Hypothetical protein F422_gp009 [*Staphylococcus* phage SA11]	61	
36	17920	17135	-	786	261	ArpR DNA-binding protein	ArpR DNA-binding protein [*Staphylococcus* phage Stau2]	77	NTP-PPase_u3 (cd11540)
37	18808	18182	-	627	208	Hypothetical protein	No significant similarity found		
38	22683	18877	-	3807	1268	Hypothetical protein	Hypothetical protein RP15_gp154 [*Staphylococcus* phage vB_Sau-RP15]	98	Beta_helix (pfam 13229)
39	22897	22751	-	147	48	Hypothetical protein	Hypothetical protein [*Staphylococcus* phage vB_SauM-HM01]	66	
40	23217	22894	-	324	107	Hypothetical protein	Hypothetical protein [*Staphylococcus* phage vB_SauM-V1SA19]	90	
41	23620	23204	-	417	138	Hypothetical protein	Hypothetical protein RP15_gp157 [*Staphylococcus* phage vB_Sau-RP15]	99	DUF3310 super family (cl13237)
42	23924	23739	-	186	61	Hypothetical protein	Hypothetical protein RP15_gp158 [*Staphylococcus* phage vB_Sau-RP15]	100	

ORF/ Region	Coordinate		Strand	Length		Putative functions	Best BLASTp match and identification	Identity (%)	Conserved protein domain family (Name and accession)
	Start	Stop		bp	aa				
43	24085	23924	-	162	53	Hypothetical protein	Hypothetical protein RP15_gp159 [*Staphylococcus* phage vB_Sau-RP15]	98	
44	26124	24085	-	2040	679	Hypothetical protein	Hypothetical protein RP15_gp160 [*Staphylococcus* phage vB_Sau-RP15]	99	
45	26466	26203	-	264	87	Hypothetical protein	Hypothetical protein RP15_gp161 [*Staphylococcus* phage vB_Sau-RP15]	100	
46	26653	26480	-	174	57	LysM domain-containing protein	LysM domain-containing protein [*Staphylococcus* phage SA11]	100	PHA02246 super family (cl10407) and LysM (cd00118)
47	27251	26628	-	624	207	Hypothetical protein	Hypothetical protein RP15_gp163 [*Staphylococcus* phage vB_Sau-RP15]	100	
48	27873	27244	-	630	209	Nucleoside 2-deoxyribosyltransferase	Hypothetical protein F422_gp183 [*Staphylococcus* phage SA11]	100	
49	30175	27875	-	2301	766	Serine/threonine protein phosphatase (EC 3.1.3.16)	Hypothetical protein MRS_052 [*Staphylococcus* phage MR003]	99	COG4639 super family (cl44097)
50	30714	30316	-	399	132	Hypothetical protein	Hypothetical protein F422_gp181 [*Staphylococcus* phage SA11]	100	

ORF/ Region	Coordinate		Strand	Length		Putative functions	Best BLASTp match and identification	Identity (%)	Conserved protein domain family (Name and accession)
	Start	Stop		bp	aa				
51	31356	30754	-	603	200	Hypothetical protein	Hypothetical protein SAC_25 [*Staphylococcus* phage SAC]	96	PHA02248 super family (cl10408)
52	31633	31370	-	264	87	Putative membrane-associated protein	Putative membrane protein [*Staphylococcus* phage StAP1]	100	
53	32070	31645	-	426	141	Ribonuclease H (EC 3.1.26.4)	Ribonuclease H [*Staphylococcus* phage StAP1]	98	RNase_H_like super family (cl14782)
54	32251	32063	-	189	62	Hypothetical protein	Hypothetical protein F422_gp176 [*Staphylococcus* phage SA11]	98	
55	32903	32265	-	639	212	Hypothetical protein	Hypothetical protein RP15_gp171 [*Staphylococcus* phage vB_Sau-RP15]	99	
56	33111	32896	-	216	71	Putative XRE-like transcriptional regulator	putative XRE-like transcriptional regulator [*Staphylococcus* phage vB_SauM-V1SA22	99	HTH_XRE (smart00530)
57	33346	33125	-	222	73	Hypothetical protein	Hypothetical protein Romulus_174 [*Staphylococcus* phage vB_SauM_Romulus]	100	
58	34206	33448	-	759	252	Lysin (EC 3.5.1.28)	Phage lysin [*Staphylococcus* phage MR003]	98	

ORF/ Region	Coordinate		Strand	Length		Putative functions	Best BLASTp match and identification	Identity (%)	Conserved protein domain family (Name and accession)
	Start	Stop		bp	aa				
59	35137	34220	-	918	305	Lysin (EC 3.5.1.28)	Lysin [*Staphylococcus* phage LSA2308]	99	PGRP (cd06583)
60	35637	35137	-	501	166	Holin	Holin [*Staphylococcus* phage SA11]	99	Phage_holin_1 super family (cl02344)
61	35905	35717	-	189	62	Hypothetical protein	Hypothetical protein F422_gp169 [*Staphylococcus* phage SA11]	100	
62	36925	36710	-	216	71	Hypothetical protein	Hypothetical protein SAC_16 [*Staphylococcus* phage SAC]	100	
63	37886	37023	-	864	287	Phage anti-repressor protein	Antirepressor protein [*Staphylococcus* phage vB_SauM-V1SA19]	100	COG3617 super family (cl34647)
64	38380	38165	-	216	71	Hypothetical protein	Hypothetical protein F422_gp166 [*Staphylococcus* phage SA11]	99	
65	38723	38391	-	333	110	Hypothetical protein	Hypothetical protein F422_gp165 [*Staphylococcus* phage SA11]	100	
66	39062	38730	-	333	110	Hypothetical protein	Hypothetical protein F422_gp164 [*Staphylococcus* phage SA11]	100	PHA02414 super family (cl10428)
67	39506	39892	+	387	128	Hypothetical protein	Hypothetical protein RP15_gp185 [*Staphylococcus* phage vB_Sau-RP15]	100	

ORF/ Region	Coordinate		Strand	Length		Putative functions	Best BLASTp match and identification	Identity (%)	Conserved protein domain family (Name and accession)
	Start	Stop		bp	aa				
68	39870	40151	+	282	93	Hypothetical protein	Hypothetical protein F422_gp162 [*Staphylococcus* phage SA11]	100	
69	40148	40561	+	414	137	Terminase small subunit	Terminase small subunit [*Staphylococcus* phage SA11]	100	
70	40578	40775	+	198	65	Terminase large subunit	Terminase large subunit [*Staphylococcus* phage SA11]	98	
71	40780	40920	+	141	46	Hypothetical protein	No significant similarity found		
72	41362	41664	+	303	100	Terminase large subunit	Terminase large subunit [*Staphylococcus* phage vB_SepM_BE05]	85	
73	41960	42328	+	369	122	Terminase large subunit	Terminase large subunit [*Staphylococcus* phage vB_SauH_SPJ2]	99	
74	42728	43018	+	291	96	Terminase large subunit	Terminase large subunit [*Staphylococcus* phage qdsa001]	80	
75	43204	44373	+	1170	389	Hypothetical protein	Hypothetical protein PHIM1EF22_0060 [*Enterococcus* phage phiM1EF22]	43	
76	44449	45102	+	654	217	Hypothetical protein	Hypothetical protein F422_gp156	95	

ORF/ Region	Coordinate		Strand	Length		Putative functions	Best BLASTp match and identification	Identity (%)	Conserved protein domain family (Name and accession)
	Start	Stop		bp	aa				
							[*Staphylococcus* phage SA11]		
77	45118	45918	+	801	266	Hypothetical protein	Hypothetical protein qdsa001_74 [*Staphylococcus* phage qdsa001]	95	
78	45905	46078	+	174	57	Hypothetical protein	Hypothetical protein RP15_gp003 [*Staphylococcus* phage vB_Sau-RP15]	91	
79	46071	46550	+	480	159	Hypothetical protein	Hypothetical protein RP15_gp004 [*Staphylococcus* phage vB_Sau-RP15]	98	
80	46593	47327	+	735	244	Putative membrane-associated protein	Putative membrane-associated protein [*Staphylococcus* phage KSAP7]	75	
81	47389	47748	+	360	119	Hypothetical protein	Hypothetical protein Romulus_010 [*Staphylococcus* phage vB_SauM_Romulus]	97	PHA02256 super family (cl10409)
82	47834	48448	+	615	204	Transposase	Hypothetical protein [Herelleviridae sp.]	91	transpos_IS607 (NF033518)
83	48423	49532	+	1110	369	Transposase	Transposase [*Staphylococcus* phage vB_SauM_Romulus]	93	guided_TnpB (NF040570)
84	49717	50082	+	366	121	Putative portal protein	Putative portal protein [*Staphylococcus* phage KSAP7]	98	Phage_portal super family (cl19194)

**ORF/ Region**	**Coordinate**		**Strand**	**Length**		**Putative functions**	**Best BLASTp match and identification**	**Identity (%)**	**Conserved protein domain family (Name and accession)**
	**Start**	**Stop**		**bp**	**aa**				
85	50087	51151	+	1065	354	Putative portal protein	Portal protein [*Staphylococcus* phage SA11]	99	
86	51309	51446	+	138	45	Transposase	Transposase [*Staphylococcus* phage vB_Sau-RP15]	86	guided_TnpB super family (cl45887)
87	51660	52364	+	705	234	Putative portal protein	portal protein [*Staphylococcus* phage SA11]	99	Phage_portal super family (cl19194)
88	52471	52668	+	198	65	Hypothetical protein	Hypothetical protein BH792_gp024 [*Staphylococcus* phage Stau2]	38	
89	52736	53500	+	765	254	Prohead protease	Prohead protease [Staphylococcus phage vB_SauM-V1SA19]	96	Peptidase_S78 super family (cl01521)
90	53514	54455	+	942	313	Hypothetical protein	Hypothetical protein LSA2308_00123 [*Staphylococcus* phage LSA2308]	88	
91	54538	55929	+	1392	463	Major capsid protein	Major capsid protein [*Staphylococcus* phage qdsa001]	95	
92	56033	56266	+	234	77	Hypothetical protein	Hypothetical protein QLX36_gp012 [*Staphylococcus* phage vB_SauM_Romulus]	99	
93	56276	57184	+	909	302	Tail fiber protein	Tail fiber protein [*Staphylococcus* phage Koomba-kaat_1]	98	
94	57202	58074	+	873	290	Hypothetical protein	Hypothetical protein RP15_gp018	93	

ORF/ Region	Coordinate		Strand	Length		Putative functions	Best BLASTp match and identification	Identity (%)	Conserved protein domain family (Name and accession)
	Start	Stop		bp	aa				
							[*Staphylococcus* phage vB_Sau-RP15]		
95	58074	58694	+	621	206	Hypothetical protein	Hypothetical protein [*Staphylococcus* phage KSAP7]	99	
96	58713	59555	+	843	280	Hypothetical protein	Hypothetical protein Koombakaat1_00174 [*Staphylococcus* phage Koomba-kaat_1]	98	
97	59548	59754	+	207	68	Hypothetical protein	Hypothetical protein F422_gp139 [*Staphylococcus* phage SA11]	100	
98	59777	61537	+	1761	586	Major tail sheath protein	Major tail sheath protein [*Staphylococcus* phage SA11]	99	
99	61600	61959	+	360	119	Hypothetical protein	Hypothetical protein [*Staphylococcus* phage vB_SauM-V1SA20]	100	
100	63251	64237	+	987	328	Hypothetical protein	Hypothetical protein [*Staphylococcus* phage vB_SauM-V1SA20]	98	
101	64285	64428	+	144	47	Hypothetical protein	Hypothetical protein [*Staphylococcus* phage vB_SauM-V1SA20]	91	
102	64465	64914	+	450	149	Hypothetical protein	Hypothetical protein F422_gp135 [*Staphylococcus* phage SA11]	98	PHA02264 (cl10410)
103	64971	65279	+	309	102	Virion structural protein	Virion structural protein	98	PHA02265 (cl10411)

ORF/ Region	Coordinate		Strand	Length		Putative functions	Best BLASTp match and identification	Identity (%)	Conserved protein domain family (Name and accession)
	Start	Stop		bp	aa				
							[*Staphylococcus* phage SA11]		
104	65464	65880	+	417	138	Hypothetical protein	Hypothetical protein RP15_gp027 [*Staphylococcus* phage vB_Sau-RP15]	99	
105	65972	66430	+	459	152	RNA polymerase beta subunit	RNA polymerase beta subunit [*Staphylococcus* phage SA11]	100	
106	66484	69480	+	2997	998	Hypothetical protein	Hypothetical protein MRS_103 [*Staphylococcus* phage StAP1]	95	
Region 1	69463	69707	+						
107	69924	70793	+	870	289	Putative tail protein	Putative tail protein [*Staphylococcus* phage vB_SauM-V1SA22]	99	LytD super family (cl27490)
108	70852	71676	+	825	274	Putative tail protein	Putative tail protein [*Staphylococcus* phage LSA2308]	95	Phage_lysozyme2 (pfam10813)
109	71728	74160	+	2433	810	Putative structural protein	Putative structural protein [*Staphylococcus* phage MR003]	98	COG3942 super family (cl34697)
110	74166	75059	+	894	297	Hypothetical protein	Hypothetical protein LSA2308_00103 [*Staphylococcus* phage LSA2308]	95	
111	75050	77506	+	2457	818	Putative glycerophosphoryl	Glycerophosphoryl diester phosphodiesterase	96	PI-PLCc_GDPD_SF super family (cl14615)

ORF/ Region	Coordinate		Strand	Length		Putative functions	Best BLASTp match and identification	Identity (%)	Conserved protein domain family (Name and accession)
	Start	Stop		bp	aa				
						Diester phosphodiesterase (EC 3.1.4.46)	[*Staphylococcus* phage vB_SauM-V1SA20]		
112	77618	78469	+	852	283	Structural protein	Structural protein [*Staphylococcus* phage vB_SauM_Romulus]	98	
113	78469	78990	+	522	173	Structural protein	Structural protein [*Staphylococcus* phage vB_SauM_Romulus]	99	
114	78990	79694	+	705	234	Baseplate protein	Baseplate protein [*Staphylococcus* phage SA11]	99	COG3628 super family (cl43917)
115	79709	79951	+	243	80	Baseplate protein	Baseplate [*Staphylococcus* phage vB_SauM-V1SA19]	84	
116	79978	80754	+	777	258	Baseplate J protein	Putative baseplate J protein [*Staphylococcus* phage KSAP7]	97	Baseplate_J super family (cl01294)
117	80766	83477	+	2712	903	Tail morphogenetic protein	Tail morphogenetic protein [*Staphylococcus* phage vB_SauM-V1SA20]	90	
118	83688	84209	+	522	173	Structural protein	Structural protein [*Staphylococcus* phage vB_SauM_Romulus]	97	
119	84232	87690	+	3459	1152	Tail protein	Tail protein [*Staphylococcus* phage vB_SauM_Romulus]	99	DUF4815 super family (cl24594)
120	87742	87912	+	171	56	Hypothetical protein	Hypothetical protein F422_gp118	100	

ORF/ Region	Coordinate		Strand	Length		Putative functions	Best BLASTp match and identification	Identity (%)	Conserved protein domain family (Name and accession)
	Start	Stop		bp	aa				
							[*Staphylococcus* phage SA11]		
121	87902	89818	+	1917	638	Capsid and scaffold protein	Capsid and scaffold protein [*Staphylococcus* phage LSA2308]	84	
122	89836	90195	+	360	119	Hypothetical protein	Hypothetical protein LSA2308_00092 [*Staphylococcus* phage LSA2308]	68	
123	90202	91566	+	1365	454	Capsid and scaffold protein	Capsid and scaffold [*Staphylococcus* phage StAP1]	88	PHA01818 super family (cl10348)
124	91650	92102	+	453	150	DNA helicase	DNA helicase [*Staphylococcus* phage qdsa001]	95	uvsW super family (cl33684)
125	92470	93654	+	1185	394	DNA helicase	DNA helicase [*Staphylococcus* phage LSA2308]	99	uvsW super family (cl33684)
126	93664	95250	+	1587	528	Rep protein	Rep protein [*Staphylococcus* phage vB_SauM_Romulus]	97	HTH super family (cl21459)
127	95243	96682	+	1440	479	Helicase/primase	Helicase/primase [*Staphylococcus* phage qdsa001]	99	41 super family (cl29348)
128	96682	96909	+	228	75	Hypothetical protein	Hypothetical protein F422_gp110 [*Staphylococcus* phage SA11]	97	
129	97011	97256	+	246	81	Hypothetical protein	Hypothetical protein BH792_gp063	88	

ORF/ Region	Coordinate		Strand	Length		Putative functions	Best BLASTp match and identification	Identity (%)	Conserved protein domain family (Name and accession)
	Start	Stop		bp	aa				
							[*Staphylococcus* phage Stau2]		
130	97266	98129	+	864	287	DNA methyltransferase	Rsrl [Staphylococcus phage vB_SepM_BE06]	88	N6_N4_Mtase (pfam01555)
131	98346	99035	+	690	229	Adenine-specific DNA-methyltransferase	Adenine-specific DNA-methyltransferase [*Staphylococcus* phage vB_Sau-RP15]	96	N6_N4_Mtase (pfam01555)
132	99105	100136	+	1032	343	Exonuclease	Exonuclease [*Staphylococcus* phage qdsa001]	94	SbcD super family (cl33866)
133	100138	100404	+	267	88	Hypothetical protein	Hypothetical protein LSA2308_00080 [*Staphylococcus* phage LSA2308]	93	PHA02275 super family (cl10412)
134	100397	102331	+	1935	644	Endonuclease (EC 2.1.1.72)	Endonuclease [*Staphylococcus* phage vB_Sau-RP15]	96	46 super family (cl33686)
135	102315	102908	+	594	197	Hypothetical protein	Hypothetical protein RP15_gp059 [*Staphylococcus* phage vB_Sau-RP15]	97	
136	102922	103998	+	1077	358	Primase	DNA Primase [*Staphylococcus* phage Koomba-kaat_1]	94	Dna super family (cl43116)
137	104059	104367	+	309	102	Hypothetical protein	Hypothetical protein F422_gp100 [*Staphylococcus* phage SA11]	98	
138	104367	104819	+	453	150	Hypothetical protein	Hypothetical protein qdsa001_138	91	PHA02277 super family (cl10413)

ORF/ Region	Coordinate		Strand	Length		Putative functions	Best BLASTp match and identification	Identity (%)	Conserved protein domain family (Name and accession)
	Start	Stop		bp	aa				
							[*Staphylococcus* phage qdsa001]		
139	105091	106353	+	1263	420	HNH endonuclease	HNH endonuclease [*Staphylococcus* phage vB_SsapH-Golestan101-M]	72	guided_IscB (NF040563)
140	106346	106963	+	618	205	Hypothetical protein	Hypothetical protein LSA2308_00072 [*Staphylococcus* phage LSA2308]	98	
141	106978	108315	+	1338	445	Ribonucleotide reductase of class Ia (aerobic), beta subunit	Ribonucleotide reductase of class Ia (aerobic), beta subunit [*Staphylococcus* phage StAP1]	99	RNRR2 (cd01049) PRK08270 super family (cl35671)
142	108331	109095	+	765	254	Ribonucleotide reductase of class Ia EC 1.17.4.1	Ribonucleotide reductase of class Ia (aerobic), alpha subunit [*Staphylococcus* phage vB_SauM-V1SA22]	99	PRK09102 super family (cl35765)
143	109413	109865	+	453	150	Ribonucleotide reductase alpha subunit EC 1.17.4.1	Ribonucleotide reductase alpha subunit [*Staphylococcus* phage PM56]	99	PRK09102 super family (cl35765)
144	110026	110382	+	357	118	Putative HNH endonuclease	HNH endonuclease family protein [*Staphylococcus* phage vB_SauM_Romulus]	93	
145	110600	111022	+	423	140	Ribonucleotide reductase of class Ia	Ribonucleotide reductase of class Ia [*Staphylococcus* phage MR003]	100	

ORF/ Region	Coordinate		Strand	Length		Putative functions	Best BLASTp match and identification	Identity (%)	Conserved protein domain family (Name and accession)
	Start	Stop		bp	aa				
146	111035	111361	+	327	108	Hypothetical protein	Hypothetical protein BH792_gp079 [*Staphylococcus* phage Stau2]	96	
147	111345	111665	+	321	106	Thioredoxin-like protein	Thioredoxin-like protein [*Staphylococcus* phage vB_SauM_Romulus]	98	Thioredoxin_like super family (cl00388)
148	111872	112474	+	603	200	Hypothetical protein	Phage protein [*Staphylococcus* phage MR003]	98	
149	112483	112785	+	303	100	DNA binding protein	DNA binding protein [*Staphylococcus* phage SA11]	96	HU_IHF super family (cl00257)
150	112863	116075	+	3213	1070	DNA polymerase I (EC 2.7.7.7)	DNA polymerase I [*Staphylococcus* phage vB_Sau-RP15]	76	DNA_pol_A (pfam00476)
151	116100	116609	+	510	169	Hypothetical protein	Hypothetical protein MRS_145 [*Staphylococcus* phage MR003]	98	
152	116621	117103	+	483	160	Hypothetical protein	Hypothetical protein QLX36_gp065 [*Staphylococcus* phage vB_SauM_Romulus]	99	
153	117174	118343	+	1170	389	Hypothetical protein	Hypothetical protein [*Staphylococcus* phage vB_SauM-V1SA22]	99	
154	118406	118630	+	225	74	Putative DNA repair protein	Putative DNA repair protein [*Staphylococcus* phage KSAP7]	97	

ORF/ Region	Coordinate		Strand	Length		Putative functions	Best BLASTp match and identification	Identity (%)	Conserved protein domain family (Name and accession)
	Start	Stop		bp	aa				
155	118960	119928	+	969	322	Putative endonuclease	Endonuclease [*Staphylococcus* phage vB_SauM_JDYN]	93	
156	120076	121002	+	927	308	Putative DNA repair recombinase	UvsX-like recombinase [*Staphylococcus* phage Koomba-kaat_1]	98	P-loop_NTPase super family (cl38936)
157	120995	121348	+	354	117	Hypothetical protein	Hypothetical protein [*Staphylococcus* phage KSAP7]	98	
158	121335	121994	+	660	219	Putative sigma factor	Putative sigma factor [Staphylococcus phage vB_SauM-V1SA19]	98	
159	122098	122730	+	633	210	Hypothetical protein	Hypothetical protein LSA2308_00052 [*Staphylococcus* phage LSA2308]	98	PHA02283 super family (cl10415)
160	122752	123264	+	513	170	Tail protein	Tail protein [*Staphylococcus* phage qdsa001]	96	Big_2 (pfam02368)
161	123281	123493	+	213	70	Putative major tail protein	Major tail protein [*Staphylococcus* phage SA11]	99	
162	123591	123845	+	255	84	Hypothetical protein	Hypothetical protein qdsa001_168 [*Staphylococcus* phage qdsa001]	90	
163	123845	124615	+	771	256	Hypothetical protein	Hypothetical protein LSA2308_00048 [*Staphylococcus* phage LSA2308]	90	PHA02284 super family (cl10416)

ORF/ Region	Coordinate		Strand	Length		Putative functions	Best BLASTp match and identification	Identity (%)	Conserved protein domain family (Name and accession)
	Start	Stop		bp	aa				
164	124608	125861	+	1254	417	Metallo-dependent phosphatase-like protein	Metallo-dependent phosphatase-like protein [*Staphylococcus* phage vB_SauM-V1SA22]	98	
165	125873	126202	+	330	109	Hypothetical protein	Hypothetical protein qdsa001_171 [*Staphylococcus* phage qdsa001]	95	
166	126275	126808	+	534	177	Hypothetical protein	Hypothetical protein LSA2308_00045 [*Staphylococcus* phage LSA2308]	98	
167	126801	127562	+	762	253	Hypothetical protein	Hypothetical protein F422_gp071 [*Staphylococcus* phage SA11]	98	sce7726_fam (NF033832)
168	127543	128043	+	501	166	Hypothetical protein	Hypothetical protein qdsa001_174 [*Staphylococcus* phage qdsa001]	89	
169	128043	128897	+	855	284	Hypothetical protein	Hypothetical protein [*Staphylococcus* phage KSAP7]	93	
170	129271	130002	+	732	243	Hypothetical protein	Hypothetical protein F422_gp068 [*Staphylococcus* phage SA11]	100	
171	130020	130478	+	459	152	Hypothetical protein	Hypothetical protein qdsa001_177 [*Staphylococcus* phage qdsa001]	95	

ORF/ Region	Coordinate		Strand	Length		Putative functions	Best BLASTp match and identification	Identity (%)	Conserved protein domain family (Name and accession)
	Start	Stop		bp	aa				
172	130543	130980	+	438	145	DNA binding protein	DNA binding protein [*Staphylococcus* phage SA11]	97	
173	130991	131626	+	636	211	Hypothetical protein	Hypothetical protein qdsa001_179 [*Staphylococcus* phage qdsa001]	95	PHA02290 super family (cl10417)
174	131662	132060	+	399	132	Hypothetical protein	Hypothetical protein QLX36_gp085 [*Staphylococcus* phage vB_SauM_Romulus]	100	PHA02291 (PHA02291)
175	132204	132455	+	252	83	Hypothetical protein	Hypothetical protein [*Staphylococcus* phage StAP1]	74	
176	132464	132727	+	264	87	Hypothetical protein	Hypothetical protein BH792_gp111 [*Staphylococcus* phage Stau2]	78	
177	132728	133012	+	285	94	Hypothetical protein	Hypothetical protein F422_gp061 [*Staphylococcus* phage SA11]	93	
178	133025	133318	+	294	97	Hypothetical protein	Hypothetical protein BH792_gp112 [*Staphylococcus* phage Stau2]	95	
179	133382	133693	+	312	103	Hypothetical protein	Hypothetical protein qdsa001_185 [*Staphylococcus* phage qdsa001]	85	
180	133698	133943	+	246	81	Hypothetical protein	Hypothetical protein BH792_gp114	95	

ORF/ Region	Coordinate		Strand	Length		Putative functions	Best BLASTp match and identification	Identity (%)	Conserved protein domain family (Name and accession)
	Start	Stop		bp	aa				
							[*Staphylococcus* phage Stau2]		
181	134079	134582	+	504	167	Hypothetical protein	Hypothetical protein SPJ221_174 [*Staphylococcus* phage vB_SauH_SPJ2]	92	
182	134605	135231	+	627	208	Pentapeptide repeat protein	Pentapeptide repeat protein [*Staphylococcus* phage vB_Sau-RP15]	64	YjbI (COG1357)
183	135250	135801	+	552	183	Hypothetical protein	Hypothetical protein BH792_gp117 [*Staphylococcus* phage Stau2]	90	
184	135807	136013	+	207	68	Hypothetical protein	Hypothetical protein [*Staphylococcus* phage StAP1]	91	
185	136013	136732	+	720	239	Pentapeptide repeat protein	Pentapeptide repeat protein [*Staphylococcus* phage vB_Sau-RP15]	92	YjbI (COG1357)
186	136719	136976	+	258	85	Hypothetical protein	Hypothetical protein SPJ221_179 [*Staphylococcus* phage vB_SauH_SPJ2]	94	
187	136970	137833	+	864	287	Hypothetical protein	Hypothetical protein F422_gp051 [*Staphylococcus* phage SA11]	78	
188	137850	138407	+	558	185	Hypothetical protein	Hypothetical protein RP15_gp107	83	

ORF/ Region	Coordinate		Strand	Length		Putative functions	Best BLASTp match and identification	Identity (%)	Conserved protein domain family (Name and accession)
	Start	Stop		bp	aa				
							[*Staphylococcus* phage vB_Sau-RP15]		
189	138506	138703	+	198	65	Hypothetical protein	1. Hypothetical protein qdsa001_194 [*Staphylococcus* phage qdsa001]	85	
190	138703	139125	+	423	140	Hypothetical protein	Hypothetical protein RP15_gp109 [*Staphylococcus* phage vB_Sau-RP15]	79	
191	139125	139856	+	732	243	Pentapeptide repeat protein	Pentapeptide repeat-containing protein [*Staphylococcus* phage PM93]	63	YjbI (COG1357)
192	139961	140260	+	300	99	Hypothetical protein	Hypothetical protein [*Staphylococcus* phage vB_SauM-V1SA22]	89	
193	141435	140944	-	492	163	Hypothetical protein	No significant similarity found		
Region 2	141502	141746	+			Repeat region			

Table S3. Intergenomic similarity of *Staphylococcus* phage vB_SauM_VL10 sequences in comparison with 30 top BLASTn hits.

Scientific Name and Accession	Query Cover (%)	E-value	Percentage identity
Staphylococcus phage vB_SauM_VL10 (OP940114.1)	100	0	100
Staphylococcus phage vB_Sau-RP15 ((MZ643272.1)	85	0	97.93
Staphylococcus phage SAC (OR234014.1)	84	0	97.74
Staphylococcus phage PBSA08 (OP856857.1)	84	0	97.74
Staphylococcus phage vB_SauM-V1SA20 (ON814135.1)	86	0	97.72
Staphylococcus phage MR003 (AP019522.1)	83	0	97.41
Staphylococcus phage StAP1 (KX532239.1)	80	0	93.7
Staphylococcus phage vB_SauM-V1SA22 (ON814136.1)	81	0	93.69
Staphylococcus phage SA11 (NC_019511.1)	84	0	93.67
Staphylococcus phage Stau2 (NC_030933.1)	79	0	93.65
Staphylococcus phage SSP49 (OQ094961.1)	83	0	93.65
Staphylococcus phage KSAP7 (LC492751.1)	79	0	93.55
Staphylococcus phage KSAP11 (LC492752.1)	79	0	93.55
Staphylococcus phage LSA2308 (MW363798.1)	80	0	93.5
Staphylococcus phage vB_SauH_SPJ2 (OQ557157.1)	79	0	93.49
Staphylococcus phage LSA2311 (ON866946.1)	80	0	93.49
Staphylococcus phage Koomba-kaat_1 (OP263969.1)	83	0	93.5
Staphylococcus phage vB_SauM_Remus (NC_022090.1)	79	0	93.17

Scientific Name and Accession	Query Cover (%)	E-value	Percentage identity
Staphylococcus phage Romulus (MW546077.1)	78	0	93.17
Silviavirus remus (MW546076.1)	79	0	93.17
Staphylococcus phage PM93 (MW546075.1)	79	0	93.17
Staphylococcus phage PM56 (MW546071.1)	78	0	93.17
Staphylococcus phage vB_SauM_Romulus (JX846613.1)	78	0	93.17
Staphylococcus phage SAP6 (OQ025230.1)	81	0	93.06
Staphylococcus phage StAP1 (OQ025229.1)	81	0	93.06
Staphylococcus phage qdsa001 (KY779848.1)	77	0	92.34
Staphylococcus phage vB_SauM-V1SA19 (ON814134.1)	79	0	92.33
Staphylococcus phage vB_SauH_DELF3 (LC576631.1)	64	0	79.67
Staphylococcus phage phiSA12 (NC_023573.1)	27	0	78.85
Staphylococcus phage vB_ScaM-V1SC04 (OP297178.1)	23	0	79.27
Staphylococcus phage Biyabeda-mokiny_1 (OP263967.1)	22	0	79.27

Table S4. Genomic features of *Staphylococcus* phage vB_SauM_VL10 and related *Silviavirus* phages investigated in this study. The percentage of identity obtained from Virus Intergenomic Distance Calculator (VIRIDIC) heat map.

Phage name and Accession number	Phage Origin	Genome Size (bp)	ORF (gene)	%GC	(%) Identity to VL 10
Staphylococcus phage vB_SauM_VL10 (OP940114.1)	Thailand	141,746	193	29.9	100
Staphylococcus phage vB_SauM-V1SA20 (ON814135.1)	France	136,866	232	30	83.869
Staphylococcus phage SAC (OR234014.1)	Republic of Korea	136,673	159	30	83.027
Staphylococcus phage PBSA08 (OP856857.1)	South Korea	136,673	119	30	83.017
Staphylococcus phage SA11 (JX194239.1)	Korea	136,326	186	30	82.947
Staphylococcus phage vB_Sau-RP15 (MZ643272.1)	Thailand	139,486	191	29.9	82.494
Staphylococcus phage MR003 (AP019522.1)	Japan	132,152	185	30	82.1
Staphylococcus phage SSP49 (OQ094961.1)	Korea	137,283	191	30	81.703
Staphylococcus phage Koomba-kaat_1 (OP263969.1)	Australia	135,469	184	30.1	81.681
Staphylococcus phage vB_SauM-V1SA22 (ON814136.1)	France	133,701	225	30	80.653
Staphylococcus phage LSA2311 (ON866946.1)	China	144,592	201	29.9	80.092

Phage name and Accession number	Phage Origin	Genome Size (bp)	ORF (gene)	%GC	(%) Identity to VL 10
Staphylococcus phage LSA2308 (MW363798.1)	China	144,592	202	29.9	80.091
Staphylococcus phage StAP1 (KX532239.1)	South Korea	135,502	192	29.85	79.967
Staphylococcus phage Stau2 (KP881332.1)	Taiwan	133,798	179	30	79.565
Staphylococcus phage vB_SauH_SPJ2 (OQ557157.1)	China	142,846	203	29.88	79.537
Staphylococcus phage StAP1 (OQ025229.1)	China	144,705	203	29.85	79.265
Staphylococcus phage SAP6 (OQ025230.1)	China	144,705	215	29.7	79.265
Staphylococcus phage vB_SauM-HM01 (OL436246.1)	South Korea	138,868	197	29.5	78.935
Staphylococcus phage vB_SauM_Romulus (JX846613.1)	Belgium	131,332	165	30	78.844
Staphylococcus phage vB_SauM_Remus (JX846612.1)	Belgium	134,643	175	30	78.79
Staphylococcus phage Romulus (MW546077.1)	Australia	136,651	186	29.9	78.698
Staphylococcus phage PM56 (MW546071.1)	Austria	136,656	185	29.9	78.694
Staphylococcus phage vB_SauM-V1SA19 (ON814134.1)	France	138,507	245	29.9	78.432

Phage name and Accession number	Phage Origin	Genome Size (bp)	ORF (gene)	%GC	(%) Identity to VL 10
Silviavirus remus (MW546076.1)	Austria	141,985	202	29.8	78.214
Staphylococcus phage PM93 (MW546075.1)	Austria	144,038	205	29.8	78.197
Staphylococcus phage KSAP11 (LC492752.1)	Japan	138,307	188	29.9	77.823
Staphylococcus phage KSAP7 (LC492751.1)	Japan	137,950	187	29.9	77.703
Staphylococcus phage qdsa001 (KY779848.1)	China	135,563	199	29.9	77.201
Staphylococcus phage vB_SauH_DELF3 (LC576631.1)	Iran	136,569	177	33.8	63.7
